# Supplementary material for: Hydrophobic interactions of FG-nucleoporins are required for dilating nuclear membrane pores into selective transport channels after mitosis
Source: Nat Struct Mol Biol. 2026 Aug 18;33(9):1354–66. doi: 10.1038/s41594-026-01865-w (PMC13561880; doi:10.1038/s41594-026-01865-w)

Extended Data Fig. 1b

Blot 1

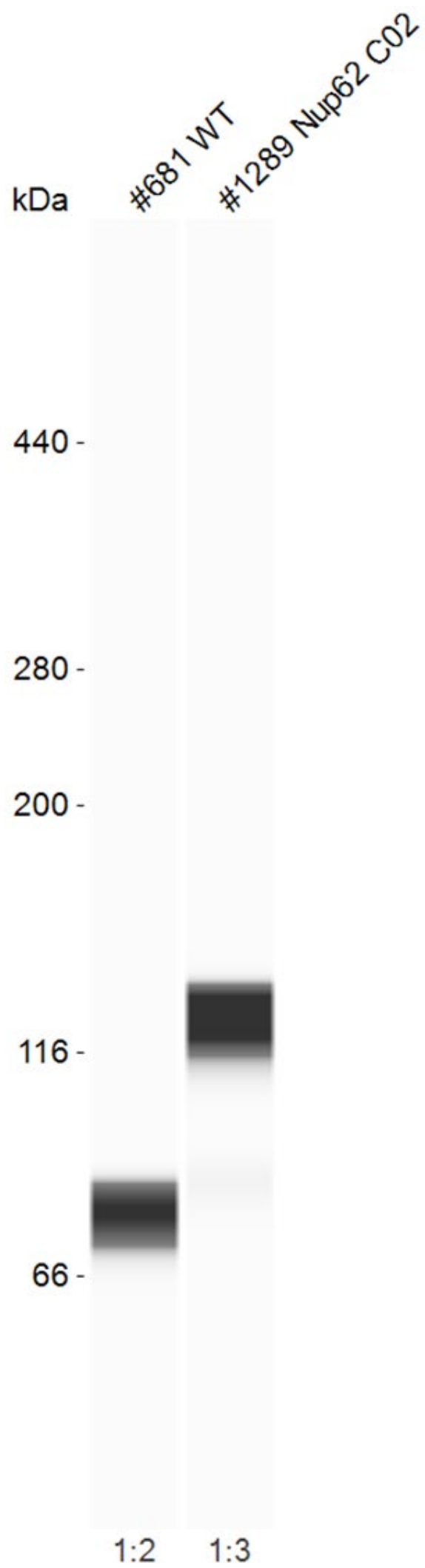

Extended Data Fig. 1b

Blot 2

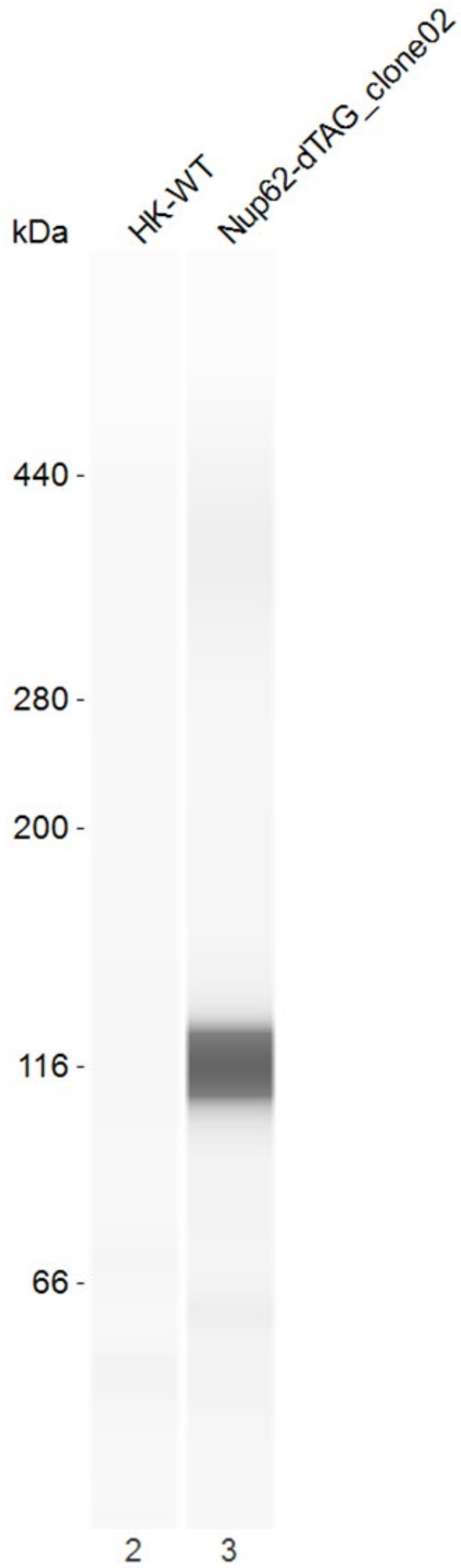

Extended Data Fig. 1b

Blot 3

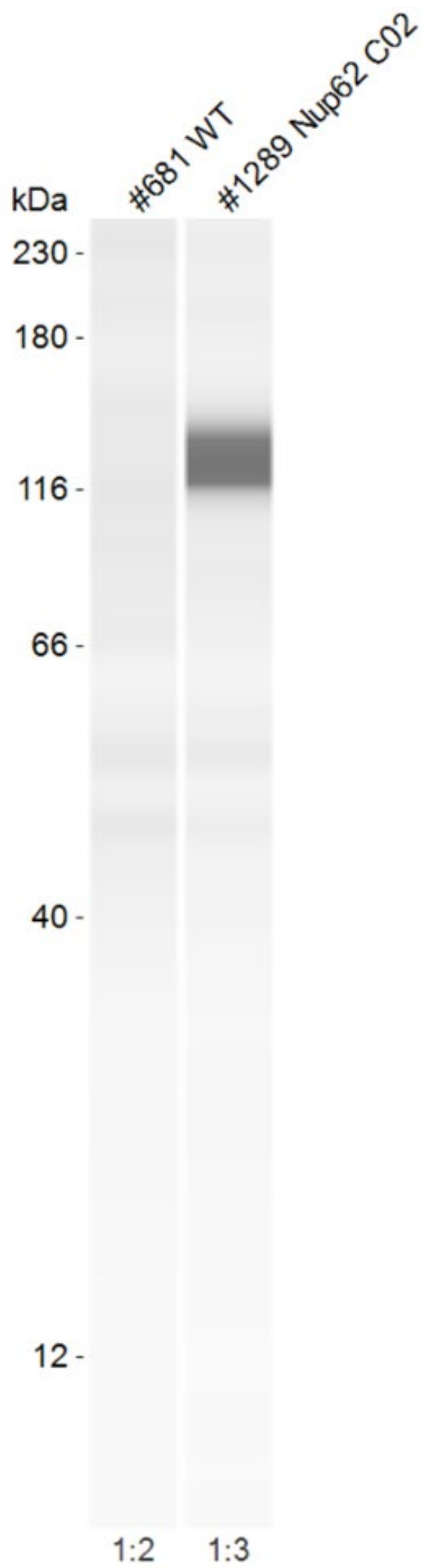

Supplement: Supplementary file 1 [file 41594_2026_1865_MOESM13_ESM.pdf]
