# Supplementary material for: Hydrophobic interactions of FG-nucleoporins are required for dilating nuclear membrane pores into selective transport channels after mitosis
Source: Nat Struct Mol Biol. 2026 Aug 18;33(9):1354–66. doi: 10.1038/s41594-026-01865-w (PMC13561880; doi:10.1038/s41594-026-01865-w)
Supplement: Supplementary file 3 [file 41594_2026_1865_MOESM1_ESM.pdf]

nature structural & molecular biology

Supplementary information https://doi.org/10.1038/s41594-026-01865-w

Hydrophobic interactions of FG-nucleoporins are required for dilating nuclear membrane pores into selective transport channels after mitosis

In the format provided by the authors and unedited

Supplementary Figures

Zhang et al. Suppl Fig. 1

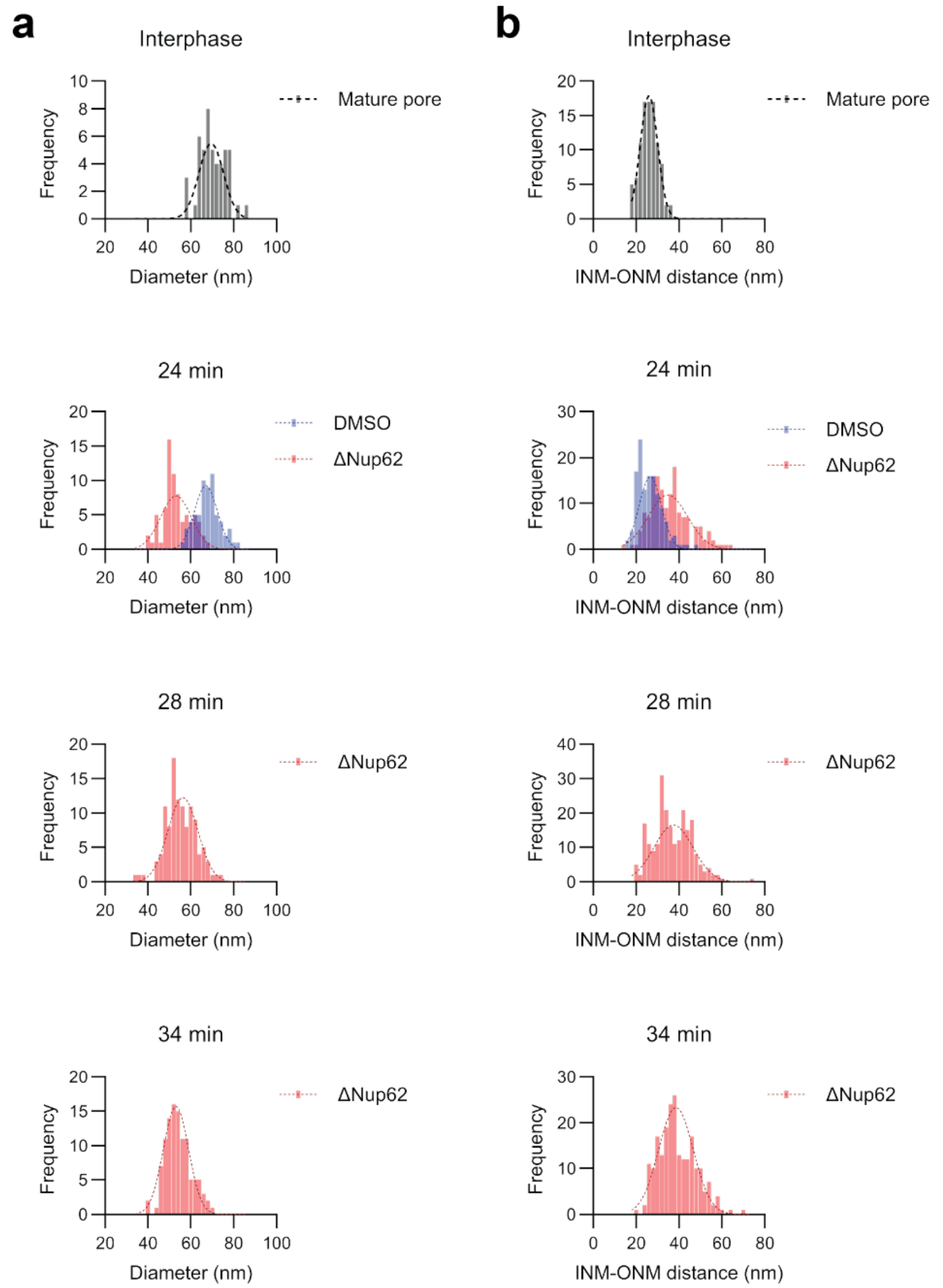

Supplementary Fig. 1 | Distribution of NPC diameter and INM-ONM distance measured in Extended Data Fig. 2E and F.

(a, b) Histograms display the distribution of NPC diameter (a) and INM-ONM distance (b) at the indicated time points after AO, corresponding to Extended Data Fig. 2e and f. Pores from interphase cells (> 180 min after AO) were analyzed for comparison (top panels). Gaussian distributions (dashed curves) were fitted to the histograms. Sample sizes are as indicated in Extended Data Fig. 2c and Fig. 1b.

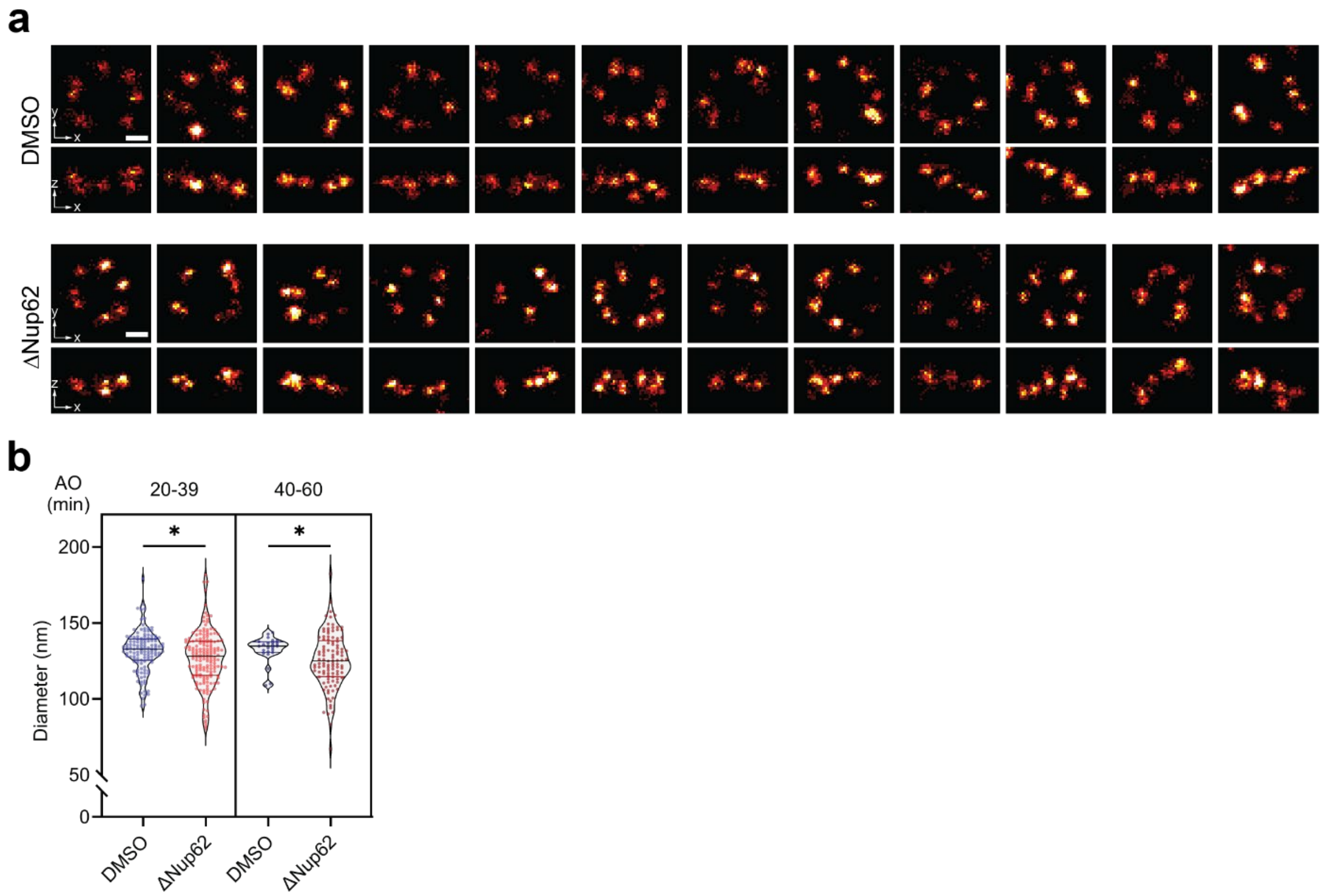

Supplementary Fig. 2 | Nup62 depletion during mitosis reduces nuclear outer ring diameter of assembled NPCs.

(a) 3D MINFLUX images (anti-Elys) of individual NPCs shown in x-y and x-z views. Images represent 2D histograms of localizations for single NPCs from HK Nup62-mEGFP-FKBP12^{F36V} cells treated with DMSO or dTAG (ΔNup62). Scale bars, 50 nm. **(b)** Subgrouping of NPC diameter measurements according to the indicated time ranges after AO, corresponding to Fig. 1g. Median and quartiles are shown as horizontal lines in the violin plot. Sample sizes: 20-39 min, DMSO, n = 126, ΔNup62, n = 167; 40-60 min, DMSO, n = 24, ΔNup62, n = 109. Statistical analyses were performed using Mann–Whitney U tests: 20-39 min, *, P = 0.0110; 40-60 min, *, P = 0.0210.

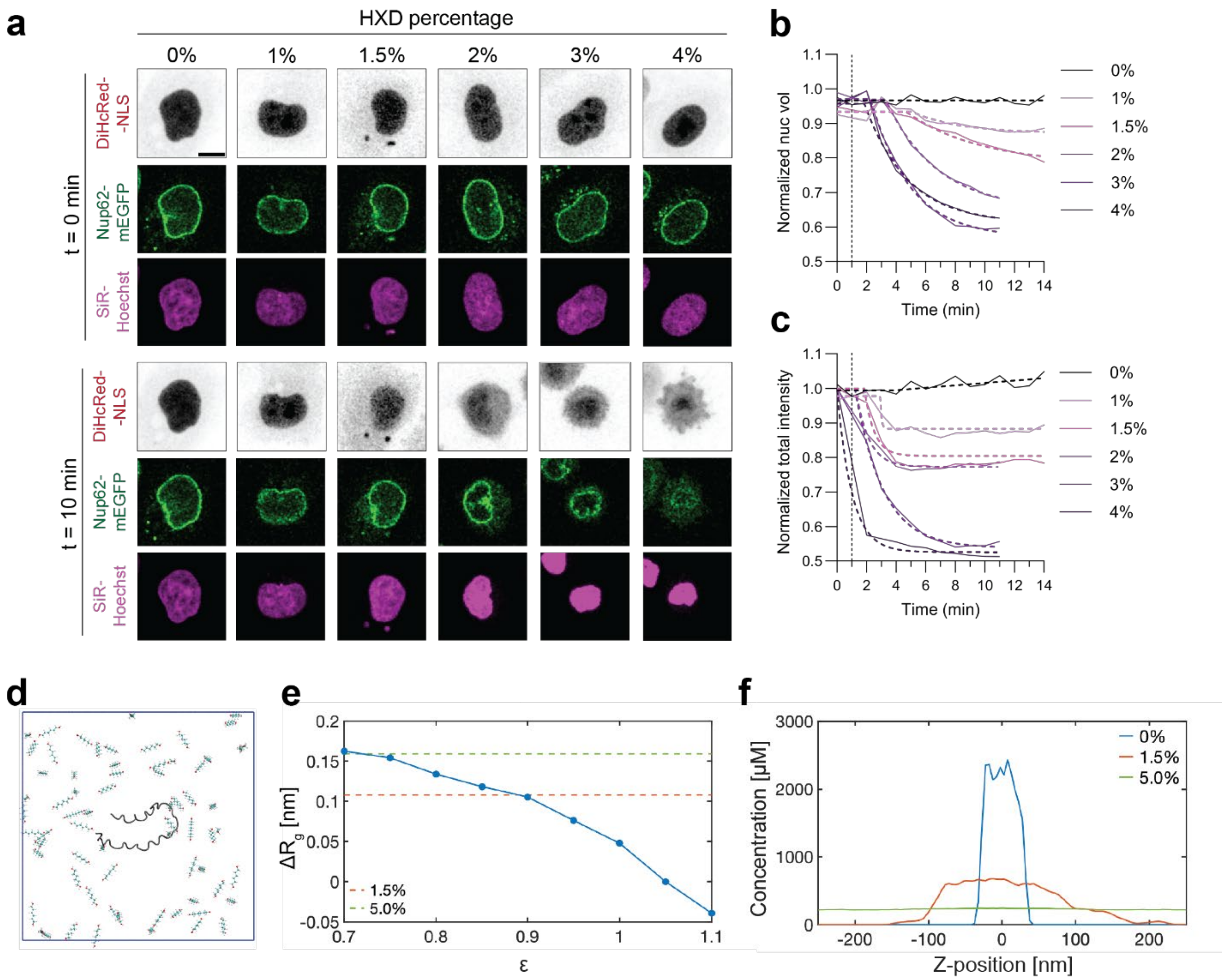

Supplementary Fig. 3 | Acute HXD treatment affects nuclear volume and nuclear import in interphase, and simulations suggest that hydrophobic interaction of Nup62-58-54 can be interrupted by HXD.

(a) HXD titration for acute disruption of FG-Nups condensate formation, assessed using the nuclear import reporter DiHcRed-NLS. HK Nup62-mEGFP-FKBP12^{F36V} cells expressing DiHcRed-NLS and stained with SiR-Hoechst were imaged just before HXD addition (t = 0 min, upper panel) and after 10 min of treatment with the indicated HXD concentrations (lower panel). Scale bar, 10 µm. **(b, c)** Kinetic plots of nuclear volume (b) and total intensity of DiHcRed-NLS within the nucleus (c), normalized to t = 0, are shown over time. HXD was added at t = 1 min, as indicated by the dashed lines. Sample sizes per condition: 0%, n = 14 cells; 1%, n = 14 cells; 1.5%, n = 10 cells; 2%, n = 12 cells; 3%, n = 11 cells; 4%, n = 12 cells. n represents independently measured cells. Data are presented as the mean. Plateau followed by one-phase decay models (dashed curves) were fitted to the kinetic data. **(d)** Image of an all-atom simulation of a Nup49

segment solvated in 1.5% HXD (protein – gray, HXD O – red, HXD C – blue, HXD H - white). Water and ions are hidden for clarity. **(e)** Plot of $\langle \Delta R_g \rangle$ (Eq. 3) as a function of ε (Eq. 1) for Nup49. Blue curve represents coarse grained simulations with different values of ε, while the dashed lines represent the values at all atom simulations with 1.5% and 5.0% HXD. **(f)** Slab density profile as a function of box length for the full length Nup62-58-54 subcomplex with specified amounts of HXD.

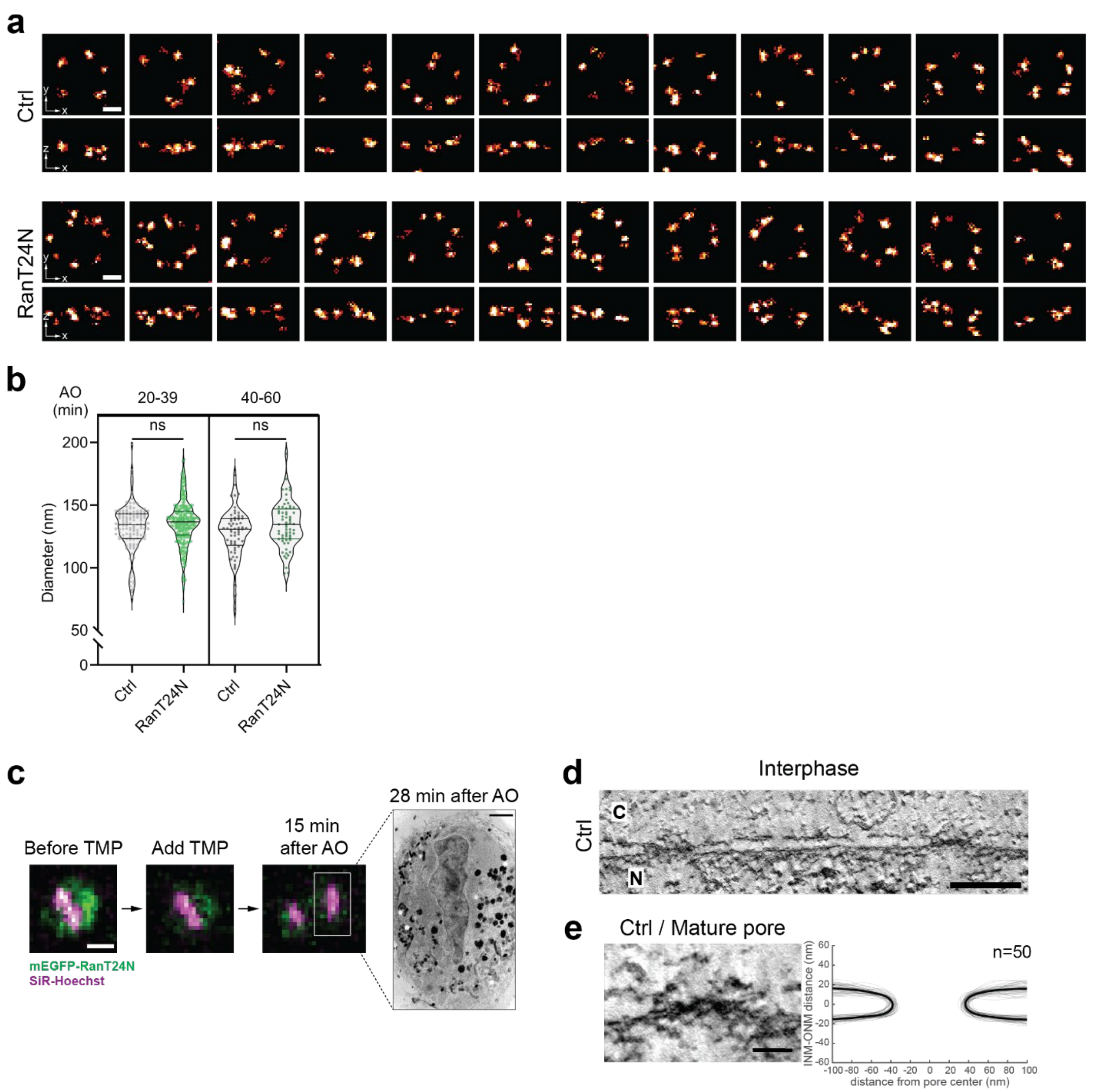

Supplementary Fig. 4 | RanT24N activation during mitotic exit does not affect NPC diameter.

(a) 3D MINFLUX images (anti-Elys) of individual NPCs shown in x-y and x-z views. Images represent 2D histograms of localizations for single NPCs from HK WT cells under control (ctrl) and acute import inhibition (RanT24N) conditions. Scale bars, 50 nm. **(b)** Subgrouping of pore diameter measurements according to the indicated division time ranges after AO, corresponding to Fig. 4d. Median and quartiles are shown as horizontal lines in the violin plot. Sample sizes: 20-39 min, Ctrl, n = 129, RanT24N, n = 276; 40-60 min, Ctrl, n = 61, RanT24N, n = 64. Statistical analyses were performed using Mann–Whitney U

tests: 20-39 min, ns, P = 0.0982; 40-60 min, ns, P = 0.0825. **(c)** Correlative live-cell and EM images of HK WT cells under RanT24N condition, analyzed by EM tomography. Cells were first imaged by light microscopy using live DNA dyes (shown in magenta) to track mitotic progression. Subsequently, 50 μM of TMP was added to induce nuclear import of mEGFP-RanT24N during mitosis. The same cells were then subjected to high-pressure freezing, resin-embedding, serial sectioning, and observed in EM. Division time after AO is indicated. Scale bars: light microscopy, 10 μm; EM, 2 μm. **(d)** Representative tomographic slice of the NE in interphase HK WT cells under Ctrl condition. N, nucleus; C, cytoplasm. Scale bar, 200 nm. (**e)** Tomographic slices showing cross-section views of nuclear pores from the same samples as in (d). Membrane profiles of all measured pores are displayed, with mean profiles highlighted in bold. Scale bar, 50 nm. Sample sizes are indicated in the figure; data were obtained from 5 independent cells with similar results.

Supplementary Tables

Supplementary Table 1: Plasmids used in this study

**Designation**	**Source or reference**
pDiHcRed-NLS	Ellenberg Group
pmScarlet-Nup58	This work
pmScarlet-Nup93	This work
pcDNA3.0_mitoLAMA-F98_IRES_mEGFP-RanT24N	This work

Supplementary Table 2: gRNA used in this study

**Target Gene**	**Designation**	**Sequence**
Nup62	Nup62C_gRNA_1-	TCGCtcaGTCAAAGGTGATCCGG

Supplementary Table 3: Antibodies used in this study

**Antibodies**	**Dilution for immune-fluorescence**	**Dilution for Simple Western**	**Source or reference**
Anti-Elys	1 to 50		Protein Atlas Antibodies, HPA031658
Anti-GFP		1 to 50	Abcam, ab290
Anti-Nup107		1 to 50	Proteintech, 19217-1-AP
Anti-Nup133	1 to 50		Abcam, ab155990
Anti-Nup153	1 to 500		Abcam, ab96462
Anti-Nup153		1 to 50	Abcam, ab245668

Anti-Nup155	1 to 100		Protein Atlas Antibodies, HPA037775
Anti-Nup188		1 to 50	Thermo Fisher, A302-322A
Anti-Nup214	1 to 100		Abcam, ab70497
Anti-Nup214		1 to 50	BETHYL, A300-716A
Anti-Nup358	1 to 100		Protein Atlas Antibodies, HPA018437
Anti-Nup54		1 to 25	Protein Atlas Antibodies, HPA035929
Anti-Nup58		1 to 25	Invitrogen, PA5-113478
Anti-Nup62		1 to 50	BD Biosciences, 610497
Anti-Nup88	1 to 50	1 to 25	BD Transduction Laboratories™, 611896
Anti-Nup98	1 to 50		Cell Signaling Technology, 2598S
Anti-Tpr	1 to 100		Protein Atlas Antibodies, HPA019661
Mab414	1 to 500	1 to 100	BioLegend, 902902
Anti-Rad21	1 to 500		Merck Millipore, 05-908
anti-Mouse-IgG, STAR 635P	1 to 250		Abberior, ST635P-1001-500UG
anti-Rabbit-IgG, STAR 580	1 to 250		Abberior, ST580-1002-500UG
anti-mouse IgG, Alexa Fluor™ 555	1:5000		Invitrogen, A-21422

Supplementary Table 4: Cell lines used in this study

**Designation**	**Source or reference**
HK WT	S. Narumiya (Kyoto University, Kyoto, Japan), RRID: CVCL_1922
HK Nup62-mEGFP-FKBP12^{F36V} #02	this work
HK Nup153-mEGFP-FKBP12^{F36V} #10-043	Brunner et al., 2025
