# Supplementary material for: Hydrophobic interactions of FG-nucleoporins are required for dilating nuclear membrane pores into selective transport channels after mitosis
Source: Nat Struct Mol Biol. 2026 Aug 18;33(9):1354–66. doi: 10.1038/s41594-026-01865-w (PMC13561880; doi:10.1038/s41594-026-01865-w)
Supplement: Supplementary file 4 [file 41594_2026_1865_MOESM2_ESM.pdf]

nature portfolio

Corresponding author(s): Jan Ellenberg

Last updated by author(s): Jun 16, 2026

Reporting Summary

Nature Portfolio wishes to improve the reproducibility of the work that we publish. This form provides structure for consistency and transparency in reporting. For further information on Nature Portfolio policies, see our Editorial Policies and the Editorial Policy Checklist.

Statistics

For all statistical analyses, confirm that the following items are present in the figure legend, table legend, main text, or Methods section.

n/a	Confirmed	
☐	☒	The exact sample size (*n*) for each experimental group/condition, given as a discrete number and unit of measurement
☐	☒	A statement on whether measurements were taken from distinct samples or whether the same sample was measured repeatedly
☐	☒	The statistical test(s) used AND whether they are one- or two-sided<br>*Only common tests should be described solely by name; describe more complex techniques in the Methods section.*
☒	☐	A description of all covariates tested
☐	☒	A description of any assumptions or corrections, such as tests of normality and adjustment for multiple comparisons
☐	☒	A full description of the statistical parameters including central tendency (e.g. means) or other basic estimates (e.g. regression coefficient) AND variation (e.g. standard deviation) or associated estimates of uncertainty (e.g. confidence intervals)
☐	☒	For null hypothesis testing, the test statistic (e.g. *F*, *t*, *r*) with confidence intervals, effect sizes, degrees of freedom and *P* value noted<br>*Give P values as exact values whenever suitable.*
☒	☐	For Bayesian analysis, information on the choice of priors and Markov chain Monte Carlo settings
☒	☐	For hierarchical and complex designs, identification of the appropriate level for tests and full reporting of outcomes
☒	☐	Estimates of effect sizes (e.g. Cohen's *d*, Pearson's *r*), indicating how they were calculated

Our web collection on statistics for biologists contains articles on many of the points above.

Software and code

Policy information about availability of computer code

Data collection	Zeiss ZEN (Carl Zeiss);<br>Abberior Imspector (v16.3.15645-m2205-win64);<br>SerialEM;<br>Compass for Simple Western (Bio-Techne).
Data analysis	CellProfiler (v4.2.5);<br>Fiji/ImageJ (v1.54p);<br>IMOD (v4.5.6);<br>GraphPad Prism (v10.5.0);<br>MINFLUX data viewer (MATLAB; https://git.embl.de/grp-eg/minflux-data-viewer);<br>Compass for Simple Western (Bio-Techne, v6.1.0);<br>Custom code (GitLab/GitHub repositories: https://git.embl.org/heriche/minflux-npc.git; https://github.com/alatham13/<br>Postmitotic_Nup62_OpenABC).

For manuscripts utilizing custom algorithms or software that are central to the research but not yet described in published literature, software must be made available to editors and reviewers. We strongly encourage code deposition in a community repository (e.g. GitHub). See the Nature Portfolio guidelines for submitting code & software for further information.

Data

Policy information about availability of data

All manuscripts must include a data availability statement. This statement should provide the following information, where applicable:

- Accession codes, unique identifiers, or web links for publicly available datasets
- A description of any restrictions on data availability
- For clinical datasets or third party data, please ensure that the statement adheres to our policy

Representative reconstructed electron tomogram data have been deposited in the Electron Microscopy Data Bank under accession code EMD-58456, and the raw tilt-series datasets have been deposited in the Electron Microscopy Public Image Archive under accession code EMPIAR-13656. Raw and preprocessed 3D MINFLUX datasets have been deposited in the BioImage Archive under accession code S-BIAD3605. Source Data are provided with this paper.

Research involving human participants, their data, or biological material

Policy information about studies with human participants or human data. See also policy information about sex, gender (identity/presentation), and sexual orientation and race, ethnicity and racism.

Reporting on sex and gender	not applicable
Reporting on race, ethnicity, or other socially relevant groupings	not applicable
Population characteristics	not applicable
Recruitment	not applicable
Ethics oversight	not applicable

Note that full information on the approval of the study protocol must also be provided in the manuscript.

Field-specific reporting

Please select the one below that is the best fit for your research. If you are not sure, read the appropriate sections before making your selection.

☒ Life sciences ☐ Behavioural & social sciences ☐ Ecological, evolutionary & environmental sciences

For a reference copy of the document with all sections, see nature.com/documents/nr-reporting-summary-flat.pdf

Life sciences study design

All studies must disclose on these points even when the disclosure is negative.

Sample size	No statistical methods were used to predetermine sample size. Sample sizes were guided by pilot experiments and standard practices in imaging-based cell biology. Sample sizes are indicated in the figure legends.
Data exclusions	Pre-specified criteria were applied uniformly: (i) live-cell/IF: imaging artifacts (out-of-focus), failed segmentations; (ii) MINFLUX data analysis: preprocessing for localizations required cfr > 0, efo first-peak only, nLoc > 5; denoising removed localizations with <10 neighbors within 20 nm; particles discarded if <50 localizations or convex-hull Feret diameter <20 nm or >200 nm, and if no robust circle fit was obtained. (iii) ET: tilt-series were excluded if reconstruction failed prior to analysis.
Replication	Units of n are reported per figure in the legend. Electron tomography analyzed ~4–8 µm² NE surface per cell across time points, yielding 156 tomograms (Nup62 depletion assay), 103 tomograms (HXD assay), and 20 tomograms (acute import inhibition assay). MINFLUX measurements were collected at multiple defined time points after anaphase onset under control and perturbations. Timelapse imaging, correlative IF, and Simple Western were performed in ≥2 independent experiments.
Randomization	Experiments were not randomized. Samples were assigned according to the indicated experimental conditions. Acquisition and sample processing were performed in parallel between conditions to minimize batch effects. Computational pipelines processed files using automated scripts in batch mode independent of condition labels.
Blinding	Investigators were not blinded during sample preparation, data acquisition, or outcome assessment. Data analysis relied on predefined thresholds and computational pipelines applied uniformly across all samples.

Reporting for specific materials, systems and methods

We require information from authors about some types of materials, experimental systems and methods used in many studies. Here, indicate whether each material, system or method listed is relevant to your study. If you are not sure if a list item applies to your research, read the appropriate section before selecting a response.

Materials & experimental systems

n/a	Involved in the study
☐	☒ Antibodies
☐	☒ Eukaryotic cell lines
☒	☐ Palaeontology and archaeology
☒	☐ Animals and other organisms
☒	☐ Clinical data
☒	☐ Dual use research of concern
☒	☐ Plants

Methods

n/a	Involved in the study
☒	☐ ChIP-seq
☒	☐ Flow cytometry
☒	☐ MRI-based neuroimaging

Antibodies

Antibodies used

Primary antibodies: anti-ELYS (Sigma-Aldrich, HPA031658), Mab414 (BioLegend, 902902), anti-GFP (Abcam, ab290), anti-Nup62 (BD Biosciences, 610497), anti-Nup58 (Invitrogen, PA5-113478), anti-Nup153 (Abcam, ab96462 / ab245668), anti-Nup107 (Proteintech, 19217-1-AP), anti-Nup133 (Abcam, ab155990), anti-Nup98 (Cell Signaling, 2598S), anti-Nup214 (Abcam, ab70497; Bethyl, A300-716A), anti-Nup155 (HPA037775), anti-Nup188 (A302-322A), anti-Nup358 (HPA018437), anti-Tpr (HPA019661), anti-Rad21 (Merck Millipore, 05-908).
Secondary antibodies: goat anti-mouse IgG Alexa Fluor 555 (Invitrogen, A-21422), anti-Mouse- IgG, STAR 635P (Abberior, ST635P-1001-500UG) ; anti-Rabbit-IgG, STAR 580 (Abberior, ST580-1002-500UG)
Secondary nanobody: anti-rabbit single-domain nanobody coupled to a DNA-PAINT sequence (Massive photonics, MASSIVE-sdAB 1-PLEX labeling kit)

Validation

Antibody specificity was supported by (i) loss-of-signal or redistribution upon acute degron-mediated depletion of target nucleoporins (e.g., Nup62-mEGFP-FKBP12F36V; Nup153-mEGFP-FKBP12F36V), (ii) expected subcellular localization at the nuclear envelope and characteristic postmitotic patterns in correlative immunofluorescence, (iii) orthogonal validation using multiple antibodies against distinct epitopes, (iv) Simple Western (Jess) detection of bands at the expected molecular weights, or (v) consistency with published literature. Negative controls and matched acquisition and processing parameters were applied across conditions.

Eukaryotic cell lines

Policy information about [cell lines and Sex and Gender in Research]

Cell line source(s)

HeLa Kyoto wild type (HK WT; RRID: CVCL_1922) was obtained from S. Narumiya (Kyoto University, Japan). CRISPR/Cas9-edited derivatives were generated in this study (HK Nup62-mEGFP-FKBP12F36V #02) and from prior work (HK Nup153-mEGFP-FKBP12F36V #10-043; Brunner et al., 2025).

Authentication

Parental identity is documented by line name and RRID: CVCL_1922 and was authenticated by sequencing. Engineered clones were confirmed by sequencing at the genomic locus, Western blot, localization analyses, and functional depletion upon dTAG treatment.

Mycoplasma contamination

Cell cultures were regularly screened for mycoplasma contamination by PCR and were consistently negative.

Commonly misidentified lines
(See ICLAC register)

No commonly misidentified cell lines were used.

Plants

Seed stocks

not applicable

Novel plant genotypes

not applicable

Authentication

not applicable
