# Supplementary material for: Hydrophobic interactions of FG-nucleoporins are required for dilating nuclear membrane pores into selective transport channels after mitosis
Source: Nat Struct Mol Biol. 2026 Aug 18;33(9):1354–66. doi: 10.1038/s41594-026-01865-w (PMC13561880; doi:10.1038/s41594-026-01865-w)
Supplement: Supplementary file 5 [file 41594_2026_1865_MOESM3_ESM.pdf]

nature portfolio

Peer Review File

Hydrophobic interactions of FG-nucleoporins are required for dilating nuclear membrane pores into selective transport channels after mitosis

Corresponding Author: Dr Jan Ellenberg

Version 0:

Decision Letter:

11th Nov 2025

Dear Dr. Ellenberg,

Thank you again for submitting your manuscript "Hydrophobic interactions of FG-nucleoporins are required for dilating nuclear membrane pores into selective transport channels after mitosis" to the journal. Please accept my apologies for the time it took to reach this decision. This was due to the fact that we needed to recruit an additional technical reviewer late in the peer review process.

We now have comments (below) from the 6 reviewers who evaluated your paper. Please note that reviewers 3 and 4 co-reviewed the manuscript. In light of those reports, we remain interested in your study and would like to see your response to the comments of the referees in the form of a revised manuscript.

You will see that while reviewers appreciate the results, they raise several concerns which will need to be addressed in a revision. Specifically, we ask that you revise the MD simulation analysis in line with comments of reviewers 3 and 4. Furthermore, reviewer 5 points out limitations of MD simulations in providing physiological insights – please address these concerns.

Another aspect which will need revision, brought up by reviewers 5 and 6, is the calculation of the pore radius. You will see that reviewer 5 suggests subtomogram averaging, but we will not editorially insist on this if their concerns can ne addressed without averaging. At minimum, we would expect a more comprehensive error analysis, as requested by both reviewers 5 and 6. In line with comments of reviewers 1 and 2, we would ask that you further explore the involvement of Nup98 and Nup93, if experimentally feasible. We agree with reviewer 2 that testing if the effect of HDX on NPC is reversible would strengthen the manuscript, if this is possible.

Please be sure to address/respond to all concerns of the referees in full in a point-by-point response and highlight all changes in the revised manuscript text file. We expect to see your revised manuscript within 3-4 months. We are committed to providing a fair and constructive peer-review process; please do not hesitate to contact us if you would like to discuss the reviews or anticipate any delay with the resubmission. If you cannot send it within this time, please contact us to discuss an extension; we will still consider your revision provided that no similar work has been accepted for publication at NSMB or published elsewhere.

REPORTING AND DATA AVAILABILITY: Please provide updated files as follows with your revision. As you already know, we put great emphasis on ensuring that the methods and statistics reported in our papers are correct and accurate.

1. REPORTING SUMMARY: Please provide an updated file:

https://www.nature.com/documents/nr-reporting-summary.pdf

This form is a dynamic ‘smart pdf’ and must be downloaded and completed in Adobe Reader.

2. REPORTING OF STRUCTURAL DATA: If there are additional or modified structures in the revision, please submit the corresponding PDB validation reports with your other files, and use the figshare integration system to provide access to maps, half-maps, and models.

Manuscripts reporting new structures should contain a table summarizing structural and refinement statistics. Please include these tables for new cryo-EM or -ET (https://www.nature.com/documents/nr-tables-cryo-em.doc and modifying this file for ET), NMR (https://www.nature.com/documents/nr-tables-nmr.doc) and X-ray crystallography (https://www.nature.com/documents/nr-tables-xray.doc). To facilitate assessment of the quality of the structural data, a stereo image of a portion of the electron density map (for crystallography papers) or of the superimposed lowest energy structures (>10; for NMR papers) should be provided with the submitted manuscript. If the reported structure represents a novel overall fold, a stereo image of the entire structure (as a backbone trace) should also be provided. For cryo-EM structures, a representative micrograph showing individual particles should be provided in the figures alongside a processing workflow (please see our Editorial for more information: https://www.nature.com/articles/s41594-025-01567-9).

DATA DEPOSITION: We require deposition of coordinates (and, in the case of crystal structures, structure factors) into the Protein Data Bank with the designation of immediate release upon publication (HPUB). Electron microscopy-derived density maps and coordinate data must be deposited in EMDB and released upon publication. Deposition and immediate release of NMR chemical shift assignments are requested. To avoid delays in publication, dataset accession numbers must be supplied with the revised manuscript and appropriate release dates must be indicated at the galley proof stage.

3. REPORTING OF LIGHT MICROSCOPY DATA: For any revision that includes light microscopy data, we ask our authors to please include a completed light microscopy reporting table [https://www.nature.com/documents/Light_microscopy_reporting_table.xlsx] to ensure the methods are described thoroughly. The table will be available to reviewers and

ultimately published, should the manuscript be accepted at the journal. When submitting the revised version of your manuscript, please pay close attention to our href="https://www.nature.com/nature-portfolio/editorial-policies/image-integrity">Digital Image Integrity Guidelines.

4. CODE AND COMPUTATIONAL WORK: If newly developed, unpublished code was used in this work, it must be provided with the revision and access to it must be disclosed in the Code Availability Statement in the manuscript. Please also provide the completed software submission checklist. This form is a dynamic ‘smart pdf’ and must be downloaded and completed in Adobe Reader, instead of opening it in a web browser: https://www.nature.com/documents/nr-software-policy.pdf

If molecular dynamics (MD) simulations were performed, please refer to this Editorial and provide a completed MD simulations checklist with your revision: https://www.nature.com/articles/s42003-023-04653-0

Lastly, if it applies, please complete and upload the completed machine learning checklist with your submission for re-review: https://www.nature.com/documents/machine-learning-checklist.pdf

5. SOURCE GEL IMAGES: Unprocessed scans should be provided for all gels and western blots presented in figures and should be clearly labelled with the figure number in the file title. The source gel images should be uncropped, unmodified images, with molecular weight markers. Uncropped, unmodified gel images from existing experimental repeats, in addition to the data shown in the figures, can also be presented in source data. Please provide these files as PDF with your revision.

Please ensure that all control panels for gels and western blots are appropriately described as loading or IP controls. Lastly, please ensure that all images in the paper are checked for duplication of panels and for splicing of gel lanes.

6. SOURCE NUMERICAL DATA: we urge authors to provide, in tabular form in excel files, the data underlying the graphical representations used in figures. This is to further increase transparency in data reporting, as detailed in this editorial (http://www.nature.com/nsmb/journal/v22/n10/full/nsmb.3110.html). Please provide one excel file per figure max, with one panel per tab. When submitting files, the title field should indicate which figure the source data pertains to.

7. DATA AVAILABILITY: this journal strongly supports public availability of data. All data used in accepted papers should be available via a public data repository, or alternatively, as Supplementary Information. If data can only be shared on request, please explain why in your Data Availability Statement and in the correspondence with your editor.

For some data types, deposition in a public repository is mandatory as detailed below:

https://www.nature.com/nature-research/editorial-policies/reporting-standards#availability-of-data

Finally, please ensure that you retain unprocessed data and metadata files after publication, ideally archiving data in

perpetuity, as these may be requested during the peer review and production process or after publication if any issues arise.

EXTENDED DATA FIGURES

When re-submitting your manuscript, please ensure that any supplementary figures and tables that are crucial to the manuscript's conclusions are converted into Extended Data figures and tables to increase visibility of these data. Extended Data figures and tables are online-only (present in the online PDF and full-text HTML versions of the paper), peer-reviewed display items that provide essential background to the article but are not included in the main article due to space constraints. A maximum of ten Extended Data display items (figures and tables) is permitted.

While we encourage the use of color in preparing figures, please note that this will incur a charge to partially defray the cost of printing. Information about color charges can be found at http://www.nature.com/nsmb/authors/submit/index.html#costs

Nature Structural & Molecular Biology is committed to improving transparency in authorship. As part of our efforts in this direction, we are now requesting that all authors identified as 'corresponding author' on published papers create and link their Open Researcher and Contributor Identifier (ORCID) with their account on the Manuscript Tracking System (MTS), prior to acceptance. This applies to primary research papers only. ORCID helps the scientific community achieve unambiguous attribution of all scholarly contributions. You can create and link your ORCID from the home page of the MTS by clicking on 'Modify my Springer Nature account'. For more information please visit please visit www.springernature.com/orcid.

Please use the link below to submit your revised manuscript and related files:

Link Redacted

Note: This URL links to your confidential home page and associated information about manuscripts you may have submitted, or that you are reviewing for us. If you wish to forward this email to co-authors, please delete the link to your homepage.

We look forward to seeing the revised manuscript and thank you for the opportunity to review your work.

Sincerely,

Katarzyna Ciazynska, PhD
(she/her)
Senior Editor
Nature Structural & Molecular Biology
https://orcid.org/0000-0002-9899-2428

Referee expertise:

Referee #1: NPC

Referee #2: NPC

Referee #3: MD simulations

Referee #4: MD simulations

Referee #5: cryo-ET, cell biology

Referee #6: imaging, MINFLUX

Reviewers' Comments:

Reviewer #1 (Remarks to the Author):

The reformation of the nuclear envelope (NE) following mitosis is intricately linked to the assembly of nuclear pore complexes (NPCs). To explore this connection, Zhang et al. very elegantly employed acute molecular perturbations in live cells with advanced imaging techniques. They found that the rapid chemical degradation of Nup62, which facilitates the

recruitment of the Nup62/Nup58/Nup54 subcomplex—a crucial component of the central channel in NPCs—interrupts NPC assembly at an intermediate stage. This disruption leads to smaller NE pores and a loss of the central transport channel, as revealed through correlative 3D electron tomography and super-resolution microscopy.
Molecular dynamics simulations suggest that 32 copies of the Nup62/Nup58/Nup54 subcomplex present in each NPC can self-associate to achieve full pore size and generate outward pressure, indicating that FG-Nup condensate formation during postmitotic NPC assembly drives pore dilation. Cells with compromised NPC assembly exhibited reduced nuclear import, smaller nuclei, and looser NE spacing. Furthermore, inhibiting nuclear import replicated these defects without affecting NPC assembly, supporting the hypothesis that nuclear import promotes nuclear expansion and consequently reduces NE spacing.
In summary, this study proposes a two-step mechanism linking NPC assembly to NE reformation: hydrophobic FG-nucleoporins first dilate the pore to its full width, enabling nuclear expansion and maintaining consistent NE spacing.
Overall, this research is highly compelling; it presents data of exceptional quality and well-controlled experiments, with a proposed model strongly supported by a diverse array of technically complementary data. The manuscript is well-written and easy to follow, with the data presented in a clear and comprehensible manner.
Specific points:
1.) The FG-Nup Nup98 plays a crucial role in establishing the permeability barrier and facilitating active transport (PMID: 21335236, PMID: 22901806). Following the depletion of Nup62, Nup98 is localized at the nuclear rim (Fig. 2 Suppl. 3D), but it may similarly contribute to the formation of FG-Nup condensates and pore dilation. It would be beneficial for the authors to include Nup98 in their simulations of condensate formation and to discuss its potential role in pore dilation – whether they think it contributes or does not contribute. This consideration could also relate to the proposed checkpoint function of Nup98 in NPC assembly (PMID: 38649536). I am not suggesting a depletion or co-depletion study involving Nup98 and Nup62, as such experiments may not be feasible within the timeframe for revision.
2.) In Fig. 3F, it states, “Note: Minor differences in NPC diameter compared to Fig. 1 reflect slight variability in shrinkage during freeze substitution.” An alternative explanation could be that the EGFP-dTAG tag on Nup62 influences NPC diameter even in untreated samples. A comparison of NPC diameters between the parental cell line and the Nup62-EGFP-dTAG line would provide valuable insights.
3.) The statement "the largest cellular compartment" (line 40) is not universally accurate; for instance, plant and yeast cells contain vacuoles that are larger than other compartments.
4.) Regarding Fig. 1 Suppl.1D, it is puzzling that degradation induced by dTAG-13 occurs more rapidly at a concentration of 250 nM than at 500 nM or 1 µM—could the authors provide clarification on this? Additionally, Fig. 1 Suppl.1E indicates differing degradation kinetics for concentrations of 100 nM and 250 nM; however, both are presented with a similar half-life of approximately 80 minutes in the bar graph, which does not align with observations from the left graph for the lower concentration.
5.) In Fig. 1 Suppl.4A, it is surprising that upon degradation of Nup62, the outer nuclear ring diameter appears smaller at time points of 40-60 minutes compared to those at around 20-39 minutes.
6.) Lines 160ff and Fig.2 Suppl.3 present important findings regarding how recruitment of Y-complex and IRC nucleoporins remains unaffected by Nup62 depletion; this information deserves equal emphasis as shown for other nucleoporins like Nup214 and Nup88 in Fig.2. The authors should consider including data on proteins such as Nup133 or Nup155 into the main figure.
7.) The recruitment patterns observed for Nup358 in Fig.2 Suppl.3 are unexpected and warrant further discussion from the authors given that the other large component of the cytoplasmic filament (Nup241/Nup88/Nup62) is absent.
8.) In Fig.2 Suppl.4, it seems that EGFP-dTAG tagging on Nup62 impacts expression levels of other nucleoporins like Nup58, Nup54, Nup88, and Nup214 even under untreated conditions when compared to wild-type cell lines; quantifying these changes would add value to understanding potential differences in NPC diameter between parental lines and those expressing Nup62-EGFP-dTAG. Also relates to point 2.
9.) Regarding lines 281ff about RanT24N's effect on NPC assembly: given that RAN-GTP gradients are known to regulate NPC assembly processes, testing whether NPC structure remains normal via ELYS staining is insightful but could be complemented by data on NPC numbers themselves—especially since reduced nuclear import may partially stem from decreased NPC numbers.
10.) line 433ff discusses speculation about FG-Nups contributing to interphase NPC assembly by promoting evagination; mentioning that FG-Nups like Nup116 can cause herniations leading to assembly defects in budding yeast may enrich this context further.
11.) The HeLa cell line is frequently abbreviated as HK, likely referring to HeLa Kyoto. It would be beneficial to mention this abbreviation earlier in the results section, so readers are informed before encountering it in the methods section.

Reviewer #2 (Remarks to the Author):

Introduction
Despite immense progress in determining the structure of the mature nuclear pore complex (NPC), many aspects of its assembly mechanism remain enigmatic. One of the key questions is which NPC components initiate the assembly and are required for the next steps to happen. Addressing these questions is not easy. On the one hand, the NPC functions on the path between transcription and translation of virtually all genes, which makes genetic interrogation very difficult. In the case of higher eukaryotes (such as mammalian cells), matters are additionally complicated by two NPC assembly modes, postmitotic and interphase assembly. In this work, the authors address the mechanism of postmitotic NPC assembly in mammalian cells using an in vivo approach based on a precisely timed chemically induced depletion of NPC components.

Key results

Specifically, this work aims to probe the role of the central channel cohesive FG-repeat nucleoporin Nup62 in postmitotic NPC assembly. To this end, the authors combine state-of-the-art imaging techniques (electron tomography and high-resolution optical imaging using DNA-PAINT and MINFUX superresolution microscopy) with advanced chemical genetic approaches to controllably and quickly deplete Nup62. By these, the authors show that removal of Nup62 through induced proteasomal degradation just prior to postmitotic NPC assembly disrupts the Nup62-containing subcomplexes Nup62-Nup54-Nup58 in the central channel and Nup214-Nup88-Nup62 in the cytoplasmic face, leading to their assembly failure and partial degradation of these Nup62-interacting NUPs.

On the ultrastructural side, the authors show that this disruption leads to a failed NPC dilation, which is accompanied by irregular spacing between the inner and outer nuclear membranes and the lack of nuclear expansion. The authors show that the latter two defects can be clearly uncoupled from the NPC assembly defect by blocking nucleocytoplasmic transport at the moment of postmitotic nuclear envelope reassembly. These results are the basis of their two-step nuclear assembly model in which NPC assembly and, consequently, active nucleocytoplasmic transport are prerequisites for nuclear expansion and nuclear membrane flattening.

A surprising result of this work is that relatively low concentrations of 1,6-hexanediol, 1.5% - a compound known to disrupt cohesive FG repeat interactions - mimic the effect of Nup62 depletion without disrupting the association of Nup62 with postmitotic NPC. All in all, this led the authors to conclude that the cohesive FG repeat interactions are a key factor in postmitotic NPC assembly. Their condensation in the central channel produces outward-pushing force that causes the NPC to dilate. In support, they provide molecular dynamic simulations of condensates formed by FG repats in Nup62-Nup54-Nup58 and Nup214-Nup88-Nup62 complexes, specifically showing that the former can produce condensates with biophysical properties (surface tension is used as a metric) capable of membrane bending.

Validity

In Figure 2 and Supplements 1- 4 the authors rigorously investigate the effect of nup62 depletion on the localization of the immediate Nup62 interactors, Nup88, Nup214, Nup58, and Nup54, as well as the more distant NUPs Nup133, Nup98 Nup155, Elys , Nup153 and Tpr. Except for the immediate interactors (Nup88, Nup214, Nup58, and Nup54) and Tpr, they observe that the NUPs generally normally localize to the postmitotic nuclear envelope. Based on these findings, they conclude that the defect in the NPC assembly is due to selective disruption of two Nup62-containing complexes (Nup62-Nup54-Nup58 and Nup214-Nup88-Nup62) and the lack of their NPC incorporation. Considering the reduced NPC diameter observed by the authors, it is curious what is happening with Nup93? Does it still localize to NPCs upon Nup62 depletion? Together with the Nup62-Nup54-Nup58 complex, Nup93 makes up the inner ring of the NPC and, intuitively, can account for the establishment of the proper NPC diameter. Its yeast ortholog Nic96 forms a coiled coil bundle with Nsp1-Nup49-Nup57 heterotrimer and acts as assembly sensor, as was shown by the Hoelz group (DOI: 10.1126/science.aac9176). Potentially, the lack of Nup93 incorporation could contribute to the NPC dilation defect.

In Figures 3A-C and Figure 3 supplement 1 authors quantitatively model the condensation propensity of Nup62-58-54 complexes (based on a pre-trained MD model) and conclude that in the confined environment of the NPC these FG repeats would form a condensate whose surface tension overcomes the threshold for membrane bending. This is speculated to generate an outward pushing force to “open” the NPC. Can it be excluded that these forces lead to a collapse rather than dilation and that it is some other mode of FG-repeat interactions that drives the dilation process? Cohesive FG repeats also interact with NTRs and the Roderick Lims group showed multiple evidence that NTRs, though their FG interactions, form an integral part of the NPC and contribute to the integrity of the nucleocytoplasmic barrier (for example, 10.1083/jcb.202108107). Can these interactions contribute to NPC dilation? In has also been reported that cohesive FG repeats bind to scaffold NUPs (10.1016/j.cell.2017.09.033) and contribute to association of cohesive FG-NUPs with the NPC and to NPC assembly. Can it be that these Velcro-type interactions make a dilated NPC a more energy-favorable state?

The authors strengthen their argument about the role of cohesive FG-repeat interactions by showing that low concentrations of 1,6-hexanediol (HDX) mimic the in vivo effect of Nup62 depletion without disrupting nup62 localization (Figure 3D-H and Supplementary 2-4). It is curious whether MD simulations can recapitulate the hexanediol effect, i.e. to reduce the surface tension below the membrane-bending limit? As HDX effects on FG condensation can be reverted (10.1016/j.cell.2007.01.044), it is also interesting whether this applies to NPC assembly.

Significance
The ultimate strength of this study is the establishment of a controllable system to quickly remove select NUPs (or even other proteins) exactly prior to mitotic exit, thus allowing a systematic analysis of the postmitotic NPC assembly mechanism and avoiding the danger of indirect effects. Perhaps such a chemogenetic strategy can also be applied to other cellular processes/factors beyond NUPs.

Although the direct role of cohesive FG interactions has yet to be more firmly established, this work contributes to our understanding of the NPC assembly showing in vivo a specific role of Nup62 in the NPC dilation step and causally linking the NPC dilation with the establishment of the nucleocytoplasmic transport function and nuclear expansion/nuclear membrane flattening. It is also highly unexpected that HDX can inhibit NPC dilation at such low concentrations. In the future, this finding may shed light on the mechanism of NPC assembly.

The work also highlights the utility of various chemogenetic strategies (the FKBP12F36V – dTAG system and LAMA) and

how they can be used to unravel complex cellular processes.

Data and methodology
The problem of postmitotic NPC assembly is addressed with adequate experimental techniques - well-characterized Nup62 depletion combined with a correlation microscopy analysis on precisely timed single cells following the anaphase onset. The authors also used an elegant way to disrupt the directional nucleocytoplasmic transport by a chemogenetically controlled release of RanT24N from mitochondrial membrane sequestration (Figure 4 and Supplements).
Although the role of cohesive FG interactions is probed with the best tools available, i.e. HDX, the mechanistic connection between cohesive FG-FG interactions and NPC dilation might be hard to draw solely from this evidence, as HDX can potentially inhibit other FG-interaction modes and even other types of binding between the NPC components.

Analytical approach
The experimental data presented in the manuscript are generally of high quality, both from the perspective of using appropriate controls and from the adequate statistical analysis of the experiments. The chemogenetic tools used for Nup62 depletion (the FKBP12F36V – dTAG system) and for controlled release of RanT24N (using the LAMA system) are thoroughly characterized. However, aside of basic “sanity checks” (see suggested improvements) this reviewer does not have adequate expertise to judge the MD aspect of this work.

Suggested improvements
Testing whether Nup93 still localizes to the NPC upon Nup62 depletion would be helpful to strengthen the argument that the Nup62-Nup54-Nup58 complex is the key factor in postmitotic NPC dilation (see validity).

It might be too much to ask, but if it is possible to pretrain the MD model in the presence of HDX it would be great to test how strongly 1.5% HDX reduces the condensate surface tension/prevents Nup62-54-58 condensate formation and if this effect can be reverted by HDX removal.

It would be great to test whether the HDX effect on the NPC assembly is reversible, i.e., whether HDX washout resolves the dilation defect as it happen in cease of cytosolic FG condensates (see validity).

As there is no guarantee that HDX is a highly specific inhibitor of FG-FG interactions, the authors shall consider toning down stating their role as the driving force of NPC dilation, discussing the possible role of alternative FG interaction modes, and explaining in more detail how mechanistically the surface tension of the FG condensates can lead to NPC dilation.

Clarity and context
The text and figures of the manuscript are well organized and easy to follow. Minor comment: in Figure 1_Supplementary 1 C-F it is not clear how the decay curves were obtained.

References
Authors should check the integrity of the citations more carefully in discussion section. For example, in lines 327-328 the citations [56,57] do not seem to match the statement they support.

Expertise note
The interpretation of molecular dynamic simulations of FG-repeat interactions is outride of this reviewer’s expertise.

Reviewer #3 (Remarks to the Author):

My main concern is the inaccurate method used to calculate surface tension in the molecular simulations, which play a central role in the researchers' main hypothesis.

The authors write, 'Molecular dynamics simulations predicted that 32 copies of the central channel subcomplex can self-associate via hydrophobic interactions to occupy the volume required for full pore size and exert an outward pushing force when recruited into the previously unoccupied pore centre.' Therefore, the simulations performed and the measurement of forces (surface tensions) play an essential role in the current hypothesis. The authors analysed the surface of the formed protein liquid droplets according to Henderson and Lekner's theory, which has previously
been used to calculate the surface tension of FUS droplets from molecular dynamics simulations. The main idea is to calculate the surface tension from the spherical space anisotropy due to thermal fluctuations. However, this requires the shape of the droplet to be dominated by actual collective effects with all shape fluctuation modes being accessible. The droplet shown in Fig. 3 has evidently far to little constituents (32!) to adopt a shape whose fluctuations justify such thermal averaging. In fact, the non-smooth droplet shape seems completely dominated by the presence of too little constituents. Consequently, the surface tension measured via this route is highly inaccurate. Fortunately, the estimation can be substantially improved by a much simpler, more accurate method that exploits the implicit solvent nature of these simulations (in contrast, Hummer performed these simulations in explicit solvent!). The authors should do the following:

The MOFF force field is an implicit solvent force field. As there is no hydrostatic pressure, the virial pressure measured

within the simulation stems solely from the interactions between the proteins (all simulation packages output box pressures). According to the virial theorem, pressure is given by: $P = 2/V_{(box)} (E_{(kin)} – Virial)$. To obtain the surface tension via this more robust mechanical route, the authors should extract the isotropic box pressures measured in the simulations performed at a constant box volume. As the box volume is largely void, these values will be very small. They should then rescale the isotropic pressure value by multiplying it by the factor $V_{(droplet)}/V_{(box)}$, where $V_{(droplet)}$ is the volume of the droplet (already estimated by the authors in their work) and $V_{(box)}$ is the chosen volume of the simulation box. This rescaling effectively compensates for the arbitrary box size. In the case of a solvent-free model only, the pressure calculated via this method is the so-called Laplace pressure (ΔP), as the hydrostatic pressure is 0 by definition in a solvent-free model and therefore $P_{(simulation)}$ equals ΔP. The surface tension can then be obtained using the formula $\Delta P = (2\sigma)/R$, where R is the average radius of the droplet (assume its spherical and resolve the effective radius from the known droplet volume) and σ is the surface tension of interest.

Reviewer #3 (Remarks on code availability):

n.a

Reviewer #4 (Remarks to the Author):

I have co-reviewed this manuscript with reviewer #3.

Reviewer #5 (Remarks to the Author):

The manuscript entitled "Hydrophobic interactions of FG-nucleoporins are required for dilating nuclear membrane pores into selective transport channels after mitosis" addresses a compelling research question in structural biology: how NPCs reform and reestablish their functional architecture during mitotic exit. Specifically, it investigates the role of the largely intrinsically disordered central channel subcomplex Nup62–58–54 in this process. The NPC is undoubtedly one of the most fascinating macromolecular assemblies, and the authors focus in this paper on the central transport channel and its intrinsically disordered FG-domains. This region is particularly challenging to characterize structurally due to its inherent disorder, yet it is indispensable for establishing the NPC's selective permeability barrier.

Overall, I find the study to be of high quality. The topic is highly interesting for NSMB and a broad scientific readership. Nevertheless, I believe the manuscript requires additional validation in some of its key results before it can meet the standard expected for publication in NSMB. One major concern relates to one of the primary experimental readouts, which are the NPC diameter measurements. These were obtained using a classical TEM sample preparation workflow including high-pressure freezing, freeze substitution, resin embedding, ultrathin sectioning, and heavy-metal staining. This approach is well known to introduce several artifacts, in contrast to the current gold standard in NPC structural research, namely cryo-FIB milling combined with cryo-ET. This raises concerns about the accuracy of the reported diameters. In addition, the MD simulations presented in the paper would benefit from closer adaptation to the native NPC geometry in order to ensure that the conclusions drawn from these analyses are robust.

In my view, the manuscript has strong potential to make a meaningful contribution to the field. Addressing the following major points would considerably strengthen the work and bring it closer to the standard expected for publication in NSMB.

Major points

In the NPC side-view images (e.g., Fig. 1B) and in the tomograms available to the reviewer, the ΔNup62 condition appears to show very intense staining within the central channel. This observation is counterintuitive, as the absence of Nup62 would be expected to result in reduced density in the central channel. What exactly is being stained in this condition? Could the strong accumulation of stain artificially bridge or "glue" the central channel, thereby preventing NPC dilation, not as a direct consequence of the loss of Nup62 but rather as an artifact of staining? This point requires clarification and should be addressed explicitly in the discussion.

Schuller et al. (Nature, 2021, "The cellular environment shapes the nuclear pore complex architecture") demonstrated using cryo-FIB and cryo-ET that the diameter of native NPCs is considerably larger than previously expected. In their study, the diameter of HeLa NPCs was measured at 111 nm, substantially greater than the 89 nm reported by von Appen et al. (Nature, 2015, "In situ structural analysis of the human nuclear pore complex") using cryo-ET and subtomogram averaging on isolated nuclear envelopes. Both of these values are markedly larger than the ~70 nm diameter reported in the present manuscript for the DMSO control condition (Fig. 1C). Notably, the cryo-FIB/cryo-ET measurements suggest a HeLa NPC diameter ~40 nm larger than that described here. From my perspective, it is highly problematic that this discrepancy is not addressed in the manuscript, and it should be explicitly discussed.

Apart from the discrepancies in diameter measurements compared to cryo-methods, a key concern is the validity of the measured diameter differences between the experimental conditions, namely the DMSO control and the ΔNup62 condition (Fig. 1B, 1C), and the interphase control and the HXD condition (Fig. 3E, 3F). The profile-based diameter measurement procedure is susceptible to several potential sources of error. For example, how precisely can the center of an individual NPC be determined, particularly when pores are slightly tilted or deviate from a circular shape? How reliably can the membrane be distinguished from the spoke ring, especially under stain-based preparation? What is the effect of knife

compression during sectioning, and to what extent do dehydration, resin polymerization, or sample pre-irradiation contribute to systematic errors in the diameter measurements?
Regarding pre-irradiation, the Methods section (line 733) reports an electron dose of 5-10 e^-/nm^2. On what basis is a sample irradiated at, for example, 10 e^-/nm^2, and should such a sample not exhibit smaller NPC diameters than one irradiated at 5 e^-/nm^2? To address these concerns, a comprehensive error analysis of the reported diameter measurements would be valuable. This should include a clear assessment of the measurement uncertainties (e.g., pore center detection, elliptical pores, pore tilt), systematic errors (e.g., knife compression, knife orientation, sample shrinkage), and the propagation of these errors. The pore diameters for each experimental condition could then be reported as $x_{measure}d$ ± uncertainty derived from the error analysis. In principle, subtomogram averaging could elegantly address some of the measurement uncertainties, as it for example inherently accounts for pore center determination and tilt. However, systematic errors in the diameter measurements would still need to be carefully quantified.

In its current form, the molecular dynamics (MD) simulation appears relatively inconclusive. One major issue is that the simulated condensate is poorly described in the manuscript. For instance, it is unclear where the α-helices of the Nup62-58-54 complex are located in the simulated condensate. Do they form an outer shell or ring around the condensate, as might be expected based on the native pore geometry, or is the simulated condensate instead a tangled cluster of α-helices and low-complexity FG domains? It could also be helpful in Fig. 3B to color the ordered and disordered domains in different colors and zoom into the assembly. How would the condensate behavior change if the linear order of residues in the FG domains were randomly permuted? Furthermore, the starting configuration of the simulation, a random placement of 32 copies of Nup62-58-54 in a box, completely neglects the cylindrical geometry of the central transport channel. From my perspective, the explanatory power of the MD simulations would be greatly improved if the starting configuration placed the 32 Nup62-58-54 copies on a ring, with their α-helices attached along the ring and the low-complexity FG domains protruding inward, modelling the more physiologically relevant arrangement in the NPC central channel. Moreover, also the comparison between the volume of the simulated condensate with the measured volume deficit (line 215-216) should include the measurement uncertainty of this quantities and discuss the large difference to the diameter values measured by cryo-FIB/cryo-ET methods.

Reviewer #6 (Remarks to the Author):

As asked by the editor, I particularly had a look at the imaging and here again the Minflux part.

To start, I want to say that the correlative imaging done is impressive. This is a major achievement and must have been hard engineering work to make it work even at a leading imaging facility. The manuscript encompasses a multitude of imaging techniques that all must be executed well in combination with modeling. In that light my comments on the imaging/Minflux are smaller detail questions. Overall the LM imaging is done well and there only some questions/clarifications that I want to raise.

It is unclear to me why 3D minflux is used, would not standard 3D PAINT/STORM work just as well? The required resolution to draw conclusions would not be compromised.

The authors use 3D minflux but never show a 3D image. There are only 2D images. Why is that? Fig 1F shows the distance of the pore from the focal plane, but not the 3D structure.
From the text it is not clear if 3D information is ever really needed/used or the 3D imaging is only used to be able to get a NPC in a non-flat membrane imaged well.
Maybe I did miss it, but how is the 3D structure of the NPC actually image and used? As with 3D minflux you will see both sides of the NPC (or is only one side formed and labelled?), is this information projected away as only the radius is used in the end?In case this information is present, it would be good to use later e.g. figure 3.
If there is 3D information, please also show a 3D image in projection of the different views to be able to judge the image quality.

uncertainty estimation of the radius. The pore radius is the most important quantity in the manuscript. There is no effort done for an error analysis here. The values are solely based on the fitted 2D radius. From the distribution of the values p-scores are computed, but certain an individual measurement is, is kept in the dark here. This could be done more in-depth. This could include with the localization precision, linkage error, projection alignment, fitting error (see also below). All of that does matter little if the differences between conditions are large compared to the diameter or if all errors are unbiased and you acquire large numbers. However, there is no discussion on this point and 5% differences in the mean might get difficult to make hard.

Details:

* Line 128/129 the average diameters are given without uncertainties. Is this below 1 nm? In any case it would be nice to state them here as this will indicate how significant the different of 126 and 133 is and the 7 vs 15 nm mentioned later in the same paragraph. From Figure 1 I would expect that this is the center of the Gaussian fit? In this case the FWHM or std could be a measure for the uncertainty to zero order.

* l628 Why is the the mean fluorescence between nucleus and cytoplasm calculated from the maximum along the optical axis? Or is the maximum projection used for segmentation? This part is a bit unclear.

L643 and following:
- The labelling with antibodies will introduce a linkage error. This is substantial compared to the localization precision. It would be good to have some comment on this.
- Why is a 100x oil immersion used for imaging into water? Z-sclaing was used of 0.67 and the recent Sau Minflux paper is referenced. But this scaling depends on the objective refractive mismatch and the depth of the imaging. Sau et al. argued that this was advice by the manufacturer but is a bit unsatisfactory. S.V. Loginov, D.B. Boltje, M.N.F. Hensgens, J.P. Hoogenboom, and E.B. van der Wee. Depth-dependent scaling of axial distances in light microscopy. Optica, 11(4):553-568, 2024. This could have some impact on lengths estimation along the optical axis - depending how structures are oriented.
- How was the particle registration done? The given reference to Evangelidis et al. is from 2014. Was their code available and directly applicable to the input data from Minflux as their application was from computer vision and not SMLM? If I remember correctly their code also needed parameters such as the number of Gaussian Mixtures but it is referenced as is?

Figure 1:
The images 1F show very good labelling density - maybe the best I have seen for Minflux images. How is the data displayed? It would be good to know how many localizations per binding site, NPC are seen on average. This was one of the largest issues with Minflux that the precision per localization is very high, but the images look poor due to missing labels. Here the images look comparable to the best NPC image with STORM/PAINT.
The shown diameter in the histograms is measured each time by fitting a radius to the correct orientation

Version 1:

Decision Letter:

Our ref: NSMB-A51614A

20th May 2026

Dear Dr. Ellenberg,

Thank you for submitting your revised manuscript "Hydrophobic interactions of FG-nucleoporins are required for dilating nuclear membrane pores into selective transport channels after mitosis" (NSMB-A51614A). It has now been seen by the original referees and their comments are below. The reviewers find that the paper has improved in revision, and therefore we'll be happy in principle to publish it in Nature Structural & Molecular Biology, pending minor revisions to satisfy the referees' final requests and to comply with our editorial and formatting guidelines.

We are now performing detailed checks on your paper and will send you a checklist detailing our editorial and formatting requirements in about 2-3 weeks. Please do not upload the final materials and make any revisions until you receive this additional information from us.

To facilitate our work at this stage, it is important that we have a copy of the main text as a word file. If you could please send along a word version of this file as soon as possible, we would greatly appreciate it; please make sure to copy the NSMB account (cc'ed above).

Thank you again for your interest in Nature Structural & Molecular Biology Please do not hesitate to contact me if you have any questions.

Sincerely,

Katarzyna Ciazynska, PhD
(she/her)
Senior Editor
Nature Structural & Molecular Biology
https://orcid.org/0000-0002-9899-2428

Reviewer #1 (Remarks to the Author):

The authors have very well addressed all the points I raised, and as far as I can judge - I am not an expert in MD simulations and MINFLUX super-resolution microscopy - the points of the other reviewers.

Reviewer #2 (Remarks to the Author):

The authors have performed substantial work to revise and improve the manuscript and have adequately addressed the points raised by this reviewer. In my opinion, the revised manuscript fulfils the high-quality standards of NSMB.

Reviewer #3 (Remarks to the Author):

The authors carefully and rigorously addressed my main comments on surface tension calculation within solvent free models. I have no further comments.

Reviewer #4 (Remarks to the Author):

I co-reviewed this manuscript with one of the reviewers who provided the listed reports. This is part of the Nature Structural & Molecular Biology initiative to facilitate training in peer review and to provide appropriate recognition for Early Career Researchers who co-review manuscripts.

Reviewer #4 (Remarks on code availability):

n.a.

Reviewer #5 (Remarks to the Author):

Regarding my previously raised concerns the authors have wholeheartedly addressed my concerns and have made substantial improvements to the manuscript. The revisions have significantly strengthened the quality and clarity of the work. From my point of view, the paper is suitable for publication in NSMB now.

Reviewer #6 (Remarks to the Author):

The authors have fully addressed my questions for clarifications and also amended them in the manuscript.
NB: Figure 1C, the yz view misses a scale bar.

Reviewer #6 (Remarks on code availability):

Partial. I had a look at the JRMPC R implementation for the fusion.

Version 2:

Decision Letter:

22nd Jul 2026

Dear Dr. Ellenberg,

We are now happy to accept your revised paper "Hydrophobic interactions of FG-nucleoporins are required for dilating nuclear membrane pores into selective transport channels after mitosis" for publication as an Article in Nature Structural & Molecular Biology.

Acceptance is conditional on the manuscript's not being published elsewhere and on there being no announcement of this work to the newspapers, magazines, radio or television until the publication date in Nature Structural & Molecular Biology.

Over the next few weeks, your paper will be copyedited to ensure that it conforms to Nature Structural & Molecular Biology style. Once your paper is typeset, you will receive an email with a link to choose the appropriate publishing options for your paper and our Author Services team will be in touch regarding any additional information that may be required.

After the grant of rights is completed, you will receive a link to your electronic proof via email with a request to make any corrections within 48 hours. If, when you receive your proof, you cannot meet this deadline, please inform us at rjsproduction@springernature.com immediately.

You will not receive your proofs until the publishing agreement has been received through our system.

Due to the importance of these deadlines, we ask that you please let us know now whether you will be difficult to contact over the next month. If this is the case, we ask you provide us with the contact information (email, phone and fax) of someone who will be able to check the proofs on your behalf, and who will be available to address any last-minute problems.

To assist our authors in disseminating their research to the broader community, our SharedIt initiative provides all co-authors with the ability to generate a unique shareable link that will allow anyone (with or without a subscription) to read the published article. Recipients of the link with a subscription will also be able to download and print the PDF.

As soon as your article is published, you can generate your shareable link by entering the DOI of your article here: http://authors.springernature.com/share. Corresponding authors will also receive an automated email with the shareable link

Note the policy of the journal on data deposition: http://www.nature.com/authors/policies/availability.html.

Your paper will be published online soon after we receive proof corrections and will appear in print in the next available issue. You can find out your date of online publication by contacting the production team shortly after sending your proof corrections.

You may wish to make your media relations office aware of your accepted publication, in case they consider it appropriate to organize some internal or external publicity. Once your paper has been scheduled you will receive an email confirming the publication details. This is normally 3-4 working days in advance of publication. If you need additional notice of the date and time of publication, please let the production team know when you receive the proof of your article to ensure there is sufficient time to coordinate. Further information on our embargo policies can be found here: https://www.nature.com/authors/policies/embargo.html

You can now use a single sign-on for all your accounts, view the status of all your manuscript submissions and reviews, access usage statistics for your published articles and download a record of your refereeing activity for the Nature journals.

If you have not already done so, we strongly recommend that you upload the step-by-step protocols used in this manuscript to the Protocol Exchange. Protocol Exchange is an open online resource that allows researchers to share their detailed experimental know-how. All uploaded protocols are made freely available, assigned DOIs for ease of citation and fully searchable through nature.com. Protocols can be linked to any publications in which they are used and will be linked to from your article. You can also establish a dedicated page to collect all your lab Protocols. By uploading your Protocols to Protocol Exchange, you are enabling researchers to more readily reproduce or adapt the methodology you use, as well as increasing the visibility of your protocols and papers. Upload your Protocols at www.nature.com/protocolexchange/. Further information can be found at www.nature.com/protocolexchange/about.

An online order form for reprints of your paper is available at https://www.nature.com/reprints/author-reprints.html. Please let your coauthors and your institutions' public affairs office know that they are also welcome to order reprints by this method.

Authors may need to take specific actions to achieve compliance with funder and institutional open access mandates. If your research is supported by a funder that requires immediate open access (e.g. according to Plan S principles or the NIH public access policy) then you should select the gold OA route, and we will direct you to the compliant route where possible. Because authors warrant under our subscription licensing terms that they haven't committed to licensing any version of their article under a licence inconsistent with the terms of our agreement – including the applicable embargo period – publication under the subscription model isn't suitable for authors whose funders require no embargo.

In approximately 10 business days you will receive an email with a link to choose the appropriate publishing options for your paper and our Author Services team will be in touch regarding any additional information that may be required.

You will not receive your proofs until the publishing agreement has been received through our system.

If you have any questions about our publishing options, costs, Open Access requirements, or our legal forms, please contact ASJournals@springernature.com

Sincerely,

Katarzyna Ciazynska, PhD
(she/her)
Senior Editor
Nature Structural & Molecular Biology
https://orcid.org/0000-0002-9899-2428

Click here if you would like to recommend Nature Structural & Molecular Biology to your librarian:
http://www.nature.com/subscriptions/recommend.html#forms

Open Access This Peer Review File is licensed under a Creative Commons Attribution 4.0 International License, which permits use, sharing, adaptation, distribution and reproduction in any medium or format, as long as you give appropriate credit to the original author(s) and the source, provide a link to the Creative Commons license, and indicate if changes were made.
In cases where reviewers are anonymous, credit should be given to 'Anonymous Referee' and the source.
The images or other third party material in this Peer Review File are included in the article's Creative Commons license, unless indicated otherwise in a credit line to the material. If material is not included in the article's Creative Commons license and your intended use is not permitted by statutory regulation or exceeds the permitted use, you will need to obtain permission directly from the copyright holder.
To view a copy of this license, visit https://creativecommons.org/licenses/by/4.0/

Point-by-point response (referee comments in blue italics, our response in black)

Reviewer #1 (Remarks to the Author):

The reformation of the nuclear envelope (NE) following mitosis is intricately linked to the assembly of nuclear pore complexes (NPCs). To explore this connection, Zhang et al. very elegantly employed acute molecular perturbations in live cells with advanced imaging techniques. They found that the rapid chemical degradation of Nup62, which facilitates the recruitment of the Nup62/Nup58/Nup54 subcomplex—a crucial component of the central channel in NPCs—interrupts NPC assembly at an intermediate stage. This disruption leads to smaller NE pores and a loss of the central transport channel, as revealed through correlative 3D electron tomography and super-resolution microscopy.

Molecular dynamics simulations suggest that 32 copies of the Nup62/Nup58/Nup54 subcomplex present in each NPC can self-associate to achieve full pore size and generate outward pressure, indicating that FG-Nup condensate formation during postmitotic NPC assembly drives pore dilation. Cells with compromised NPC assembly exhibited reduced nuclear import, smaller nuclei, and looser NE spacing. Furthermore, inhibiting nuclear import replicated these defects without affecting NPC assembly, supporting the hypothesis that nuclear import promotes nuclear expansion and consequently reduces NE spacing.

In summary, this study proposes a two-step mechanism linking NPC assembly to NE reformation: hydrophobic FG-nucleoporins first dilate the pore to its full width, enabling nuclear expansion and maintaining consistent NE spacing. Overall, this research is highly compelling; it presents data of exceptional quality and well-controlled experiments, with a proposed model strongly supported by a diverse array of technically complementary data. The manuscript is well-written and easy to follow, with the data presented in a clear and comprehensible manner.

We sincerely thank the reviewer for this positive and thoughtful evaluation of our work. We greatly appreciate the recognition of the conceptual advance, the technical rigor, and the clarity of presentation of our manuscript.

Specific points:

1.) The FG-Nup Nup98 plays a crucial role in establishing the permeability barrier and facilitating active transport (PMID: 21335236, PMID: 22901806). Following the depletion of Nup62, Nup98 is localized at the nuclear rim (Fig. 2 Suppl. 3D), but it may similarly contribute to the formation of FG-Nup condensates and pore dilation. It would be beneficial for the authors to include Nup98 in their simulations of condensate formation and to discuss its potential role in pore dilation – whether they think it contributes or does not contribute. This consideration could also relate to the proposed checkpoint function of Nup98 in NPC assembly (PMID: 38649536). I am not suggesting a depletion or co-depletion study involving Nup98 and Nup62, as such experiments may not be feasible within the timeframe for revision.

We thank the reviewer for this insightful and forward-looking suggestion. We fully agree that Nup98 is a key FG-nucleoporin with well-established roles in permeability barrier formation and active transport, and its potential contribution to condensate behavior during postmitotic NPC assembly is an important consideration.

Importantly, our experimental system provides a functional separation between Nup98 presence and pore dilation. Upon acute degradation of Nup62, Nup98 remains detectable at the nuclear rim (Fig. 2 Suppl. 3D, revised Extended Data Fig. 7C), yet pore dilation fails to occur. Thus, the presence of Nup98 is not sufficient to drive full pore expansion in the absence of the Nup62 subcomplex. This observation supports the conclusion that the central channel subcomplex contributes to pore dilation in a manner that is distinct from and non-redundant to Nup98. We therefore focused our simulations on the Nup62 subcomplexes, as they are the module we acutely removed in our experimental system and the component whose loss accounts for the reduced pore diameter.

We agree that a potential additional contribution of Nup98 in addition to the Nup62 complex is an intriguing possibility. A combined and selective perturbation strategy that allows to explore the function of both and if these functions interact together with extended simulations incorporating both FG components would provide a comprehensive framework to address this question. However, such new experimental and theoretical analyses would unfortunately go far beyond the scope of revising the present study, which is focused on defining the molecular and biophysical role of hydrophobic interactions within the Nup62 subcomplexes in driving pore dilation during postmitotic NPC maturation.

To address the reviewer's suggestion, we have now expanded the Discussion (line 383) to explicitly consider the potential contribution of additional FG-Nups, including Nup98, to pore dilation during NPC maturation.

2.) In Fig. 3F, it states, "Note: Minor differences in NPC diameter compared to Fig. 1 reflect slight variability in shrinkage during freeze substitution." An alternative explanation could be that the EGFP-dTAG tag on Nup62 influences NPC diameter even in untreated samples. A comparison of NPC diameters between the parental cell line and the Nup62-EGFP-dTAG line would provide valuable insights.

We thank the reviewer for this important control consideration. We fully agree that it is essential to exclude the possibility that the tagging on Nup62 influences NPC diameter under basal conditions.

To directly address this point, we performed parallel high-pressure freezing and freeze substitution of the parental (HK WT) cell line and the HK Nup62-mEGFP-FKBP^{F36V} knock-in line under identical conditions and processed them simultaneously within the same batch. Quantitative analysis revealed no significant difference in NPC diameter between the two cell lines. The corresponding analysis is provided below for the reviewer's information.

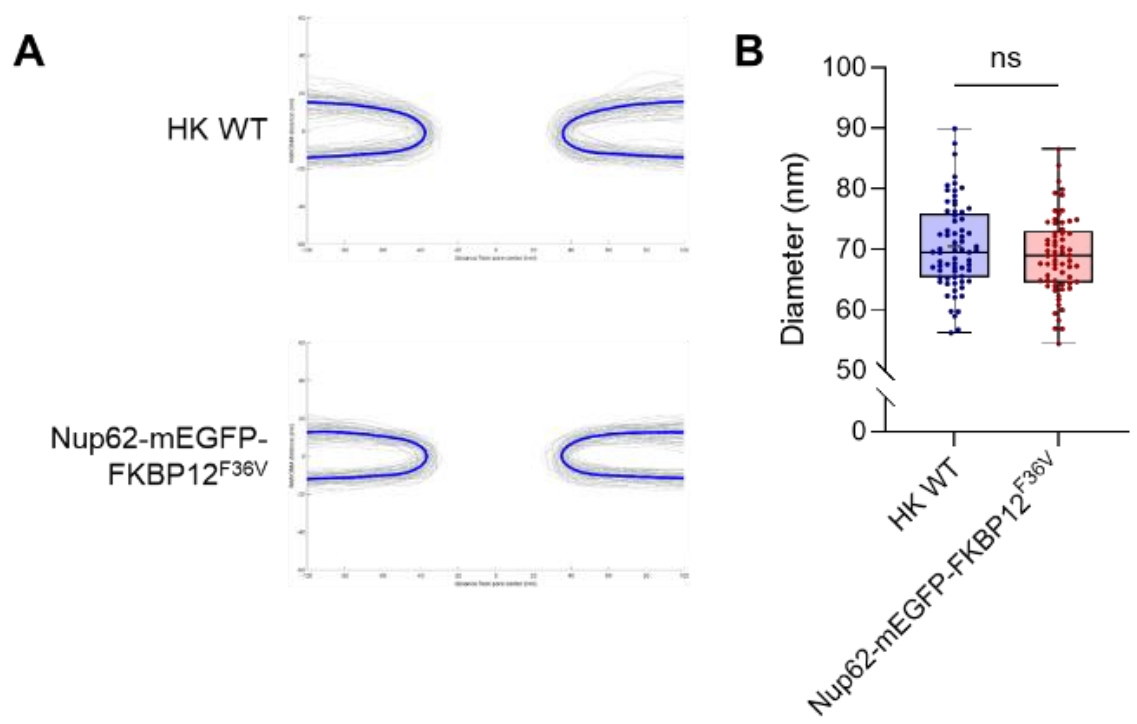

Figure Legend: (**A**) Membrane profiles of all measured pores from HK WT (n=69) and Nup62-mEGFP-FKBP^{F36V} (n=65) interphase cells are displayed, with mean profiles highlighted in bold. (**B**) Box-and-whisker plot quantifying NPC diameters derived from (A). The median, minimum to maximum range, and all individual data points are displayed. Welch's two-tailed t-test analysis indicates no significant difference (ns).

These results demonstrate that the mEGFP-FKBP^{F36V} tag does not measurably affect pore size under basal conditions. The minor differences noted between Fig. 1 and Fig. 3 therefore reflect small batch-to-batch variability inherent to independent freeze substitution experiments rather than genotype-dependent effects. We note this effect and can exclude that it comes from influence of the degron tag itself; we have now clarified this point in the methods section of the manuscript (line 801-804), but would suggest not to include this data in the manuscript.

3.) The statement "the largest cellular compartment" (line 40) is not universally accurate; for instance, plant and yeast cells contain vacuoles that are larger than other compartments.

We thank the reviewer for pointing this out. We agree that this phrasing is not universally valid across eukaryotes.

We have therefore revised the text (line 40-42) to avoid overgeneralization.

4.) Regarding Fig. 1 Suppl.1D, it is puzzling that degradation induced by dTAG-13 occurs more rapidly at a concentration of 250 nM than at 500 nM or 1 µM—could the authors provide clarification on this? Additionally, Fig. 1 Suppl.1E indicates differing degradation kinetics for concentrations of 100 nM and 250 nM; however, both are presented with a similar half-life of approximately 80 minutes in the bar graph, which does not align with observations from the left graph for the lower concentration.

We thank the reviewer for carefully examining the kinetics data.

First, regarding the non-monotonic concentration dependence observed in Fig. 1 Suppl. 1D (revised Extended Data Fig. 1D), the finding that 250 nM dTAG-13 induces more rapid degradation than 500 nM or 1 µM is consistent with the original characterization of dTAG-13 (DOI: 10.1038/s41589-018-0021-8, Fig. 2a), in which reduced degradation efficiency at higher concentrations relative to intermediate doses is evident. Such behavior is characteristic of heterobifunctional degraders and is attributed to concentration-dependent effects on productive ternary complex formation, where excessive compound can impair efficient target engagement and degradation (DOI: 10.1021/acsbiomedchemau.2c00037). Thus, the observed concentration dependence aligns with previously described properties of this kind of degrader.

Second, concerning the apparent discrepancy between the curves at 100 nM and 250 nM (Fig. 1 Suppl. 1E, revised Extended Data Fig. 1E) and the similar half-lives shown in the bar graph, this difference primarily reflects variation in the extent of degradation rather than in the exponential rate constant. Half-lives were derived using a global one-phase exponential decay model with a

free plateau (GraphPad Prism), treating each replicate measurement as an individual data point. The fitted rate constants are comparable for 100 nM (K = 0.00875 min^{-1}; t½ = 79.2 min) and 250 nM (K = 0.00953 min^{-1}; t½ = 72.7 min), indicating similar degradation kinetics.

However, the fitted plateau differs substantially between conditions (0.57 at 100 nM versus 0.12 at 250 nM), indicating incomplete degradation at the lower concentration. This reduced amplitude creates the visual impression of slower kinetics at 100 nM, even though the exponential rate constants are similar.

5.) In Fig. 1 Suppl.4A, it is surprising that upon degradation of Nup62, the outer nuclear ring diameter appears smaller at time points of 40-60 minutes compared to those at around 20-39 minutes.

We thank the reviewer for this careful observation.

To directly address this point, we quantitatively compared nuclear outer ring diameters between the two-time windows (20-39 min and 40-60 min). The mean diameters after Nup62 degradation were 126.8 nm (95% CI: [124.1 nm, 129.5 nm]) and 125.4 nm (95% CI: [122.0 nm, 128.8 nm]), respectively. A two-tailed t-test revealed no statistically significant difference between the groups, indicating that pore diameter remains stable across these intervals.

Thus, the apparent visual difference reflects sampling variability rather than progressive constriction of the nuclear outer ring. For clarity and transparency, we provide the corresponding quantitative comparison plot below.

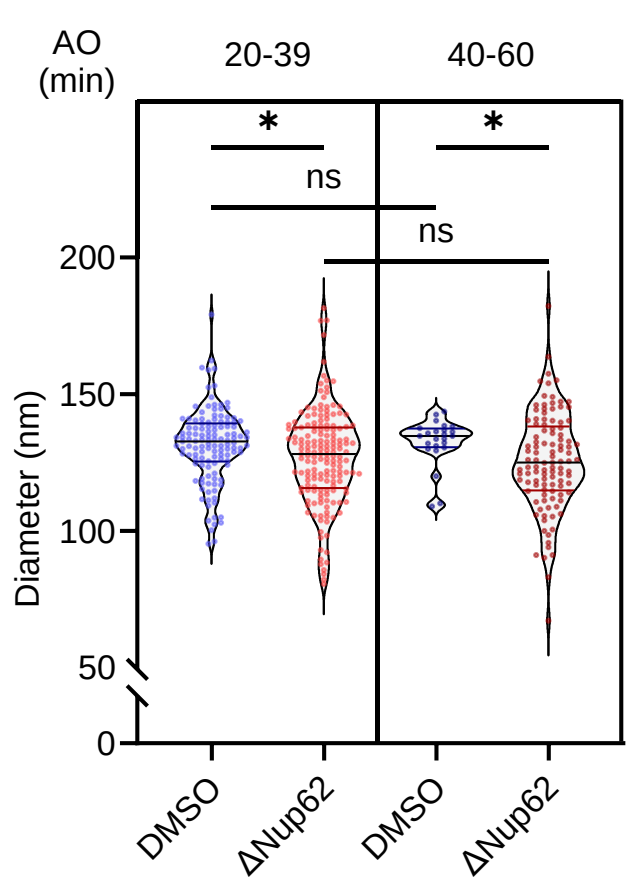

Figure Legend: Subgrouping of NPC diameter measurements according to the indicated time ranges after AO. Median and quartiles are shown in the violin plot. Two-tailed Mann-Whitney test is used for the statistical analysis. Statistical analysis was performed using a two-tailed Mann-Whitney test. Statistical significance applies: *, P ≤ 0.05; **, P ≤ 0.01; ***, P ≤ 0.001; ****, P ≤ 0.0001; ns, not significant.

6.) Lines 160ff and Fig.2 Suppl.3 present important findings regarding how recruitment of Y-complex and IRC nucleoporins remains unaffected by Nup62 depletion; this information deserves equal emphasis as shown for other nucleoporins like Nup214 and Nup88 in Fig.2. The authors should consider including data on proteins such as Nup133 or Nup155 into the main figure.

We thank the reviewer for this constructive suggestion. We agree that demonstrating preserved recruitment of outer and/or inner ring complex components is critical for defining the assembly stage at which NPC maturation is arrested upon Nup62 depletion.

In response, we have revised Fig. 2 to include Nup133 as a representative outer ring component in the main figure, together with newly included data for inner ring component Nup93, and reorganized the corresponding supplementary data (Fig. 2 Suppl. 3, revised Extended Data Fig.

7). These changes strengthen the presentation by more clearly distinguishing intact scaffold assembly from defective central channel maturation.

7.) The recruitment patterns observed for Nup358 in Fig.2 Suppl.3 are unexpected and warrant further discussion from the authors given that the other large component of the cytoplasmic filament (Nup241/Nup88/Nup62) is absent.

We thank the reviewer for highlighting this important point.

The preserved recruitment of Nup358 is consistent with the recently resolved high-resolution cryo-EM structures of the human NPC (DOIs: 10.1126/science.abm9129, 10.1126/science.abm9506, 10.1126/science.abl8280), which show that Nup358 is anchored to the cytoplasmic face of the Y-complex. There, it pentamerizes and functions as an architectural element of the cytoplasmic ring, stabilizing interactions between Y-complexes rather than directly associating with the Nup62-containing subcomplexes.

Accordingly, the retention of Nup358 following Nup62 depletion aligns with its structural positioning within the outer ring scaffold. In contrast, Nups that directly or functionally associate with the Nup62 are selectively affected, consistent with our central finding that Nup62 depletion specifically disrupts Nup64-58-54 and Nup214-88-62 subcomplex maturation rather than scaffold assembly.

We have revised the Discussion section (line 353-355) to clarify this structural rationale.

8.) In Fig.2 Suppl.4, it seems that EGFP-dTAG tagging on Nup62 impacts expression levels of other nucleoporins like Nup58, Nup54, Nup88, and Nup214 even under untreated conditions when compared to wild-type cell lines; quantifying these changes would add value to understanding potential differences in NPC diameter between parental lines and those expressing Nup62-EGFP-dTAG. Also relates to point 2.

We thank the reviewer for this careful observation.

To directly address this point, we quantitatively reanalyzed our Simple Western data and compared the expression levels of Nup58, Nup54, Nup88, and Nup214 under untreated conditions between the parental (HK WT) and Nup62-mEGFP-FKBP^{F36V} knock-in cell lines. Protein levels were normalized to total loaded protein amount detected by the Jess ProteinSimple normalization module and subsequently normalized to the parental control. This analysis revealed no significant differences in expression levels between the two lines under basal conditions. The corresponding quantification is provided below for transparency.

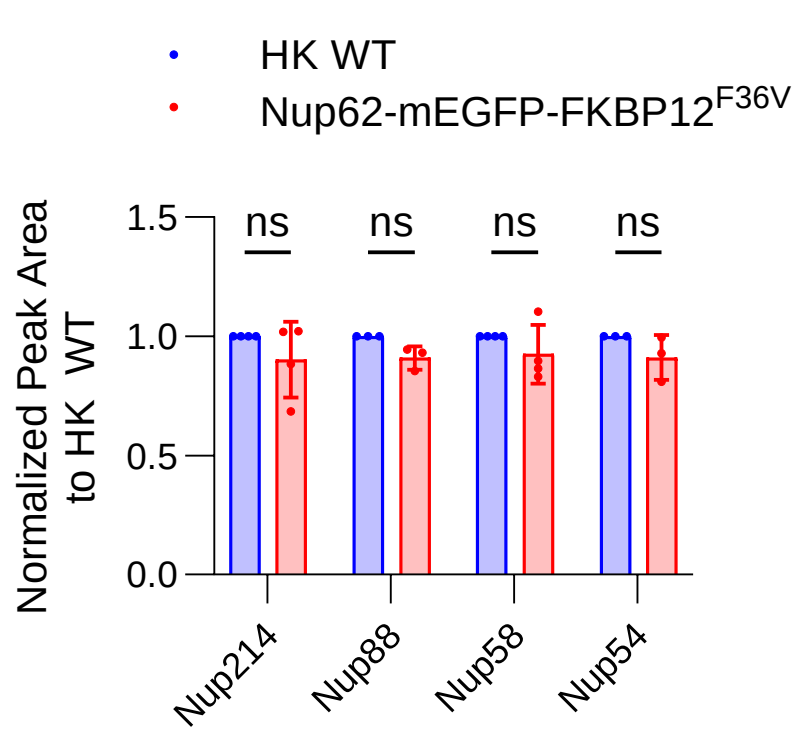

Figure Legend: Quantification of normalized protein levels of Nups detected by Simple Western analysis of protein extracts from the HK WT and HK Nup62-mEGFP-FKBP^{F36V} cell lines treated under DMSO conditions. Protein levels were first normalized to total protein content and then further normalized relative to HK WT. Multiple unpaired t-test analysis indicates no significant difference (ns).

The apparent differences in band intensity in the displayed blots most likely arise from minor loading variability inherent to the assay. Although equal volumes of lysate (0.4 µg/µl), diluted from concentrated cell extracts, were loaded, total protein detection indicates small variations in the actual amount of protein loaded across samples (see below). These differences are accounted for during normalization and do not reflect genuine differences in protein expression.

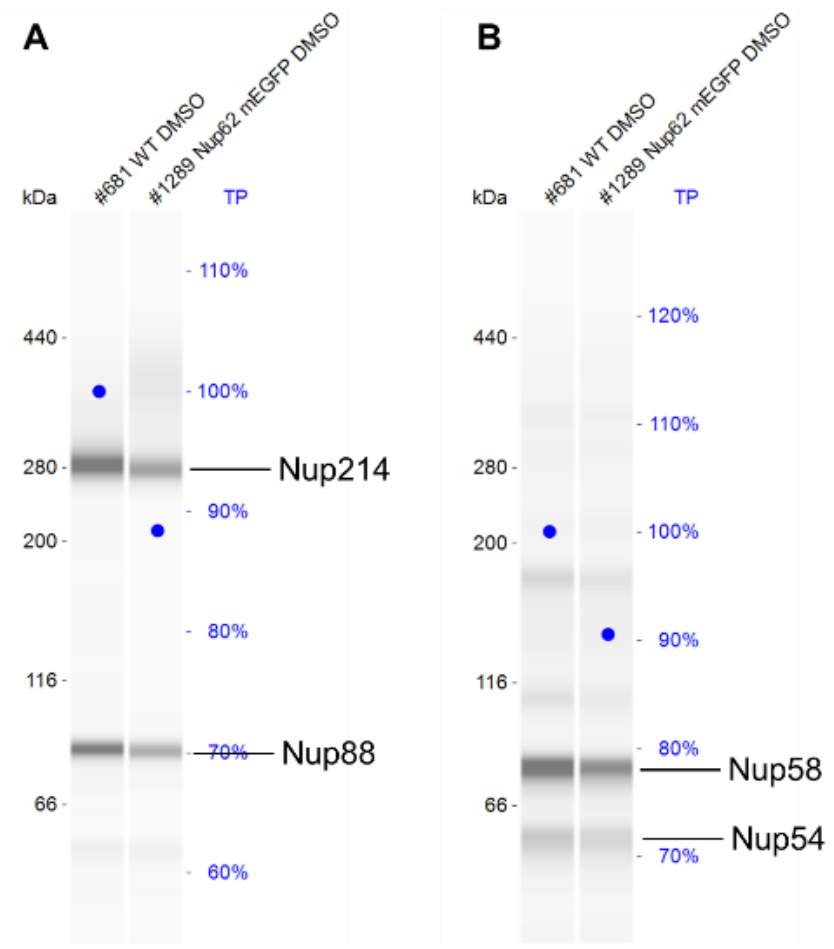

Figure Legend: Simple Western analysis of protein extracts from the HK WT and HK Nup62-mEGFP-FKBP^{F36V} cell lines treated under DMSO conditions. Antibodies were used in the following order: left panel, anti-Nup214 & anti-Nup88; right panel, anti-Nup58 & anti-Nup54. TP indicates relative total protein levels.

We have revised the Figure Legend section (line 1536-1537) to clarify this point.

9.) Regarding lines 281ff about RanT24N's effect on NPC assembly: given that RAN-GTP gradients are known to regulate NPC assembly processes, testing whether NPC structure remains normal via ELYS staining is insightful but could be complemented by data on NPC numbers themselves—especially since reduced nuclear import may partially stem from decreased NPC numbers.

We thank the reviewer for this important point.

To directly address this possibility, we quantified NPC density under RanT24N and control conditions using our correlative electron tomography data. This analysis reveals no reduction in NPC number from RanT24N condition compared to Ctrl condition, indicating that the observed decrease in nuclear import is not due to reduced pore abundance. This result is consistent with our structural analysis, which shows that ELYS-positive assembled NPCs are present at comparable density in both conditions (Fig. 4C). The corresponding quantification is provided below for transparency.

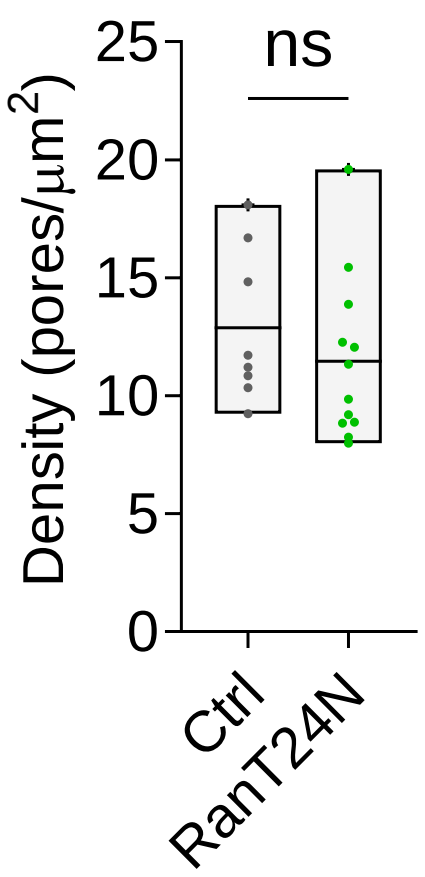

Figure Legend: Density of nuclear pores in HK WT cells under acute import inhibition (RanT24N) at 28 min after AO, quantified from correlative ET imaging. Interphase Ctrl cells, lacking mito-LAMA and mEGFP-RanT24N expression but treated identically, were used for comparison. Welch's two-tailed t-test analysis indicates no significant difference (ns).

10.) line 433ff discusses speculation about FG-Nups contributing to interphase NPC assembly by promoting evagination; mentioning that FG-Nups like Nup116 can cause herniations leading to assembly defects in budding yeast may enrich this context further.

We thank the reviewer for this valuable suggestion. We agree that studies in budding yeast demonstrating that perturbation of FG-Nups such as Nup116 leads to NE herniations associated with NPC assembly defects provide important conceptual context for our discussion of membrane remodeling regulated by FG-rich domains.

We have incorporated this example into the Discussion (line 465-468) to broaden the evolutionary and mechanistic framework and to highlight that FG-Nups may influence nuclear membrane topology during NPC assembly.

11.) The HeLa cell line is frequently abbreviated as HK, likely referring to HeLa Kyoto. It would be beneficial to mention this abbreviation earlier in the results section, so readers are informed before encountering it in the methods section.

We thank the reviewer for this helpful suggestion. We have now defined HeLa Kyoto (HK) at its first mention in the Results section (line 105) to ensure that readers are familiar with the abbreviation before it appears in the Methods.

Reviewer #2 (Remarks to the Author):

Introduction

Despite immense progress in determining the structure of the mature nuclear pore complex (NPC), many aspects of its assembly mechanism remain enigmatic. One of the key questions is which NPC components initiate the assembly and are required for the next steps to happen. Addressing these questions is not easy. On the one hand, the NPC functions on the path between transcription and translation of virtually all genes, which makes genetic interrogation very difficult. In the case of higher eukaryotes (such as mammalian cells), matters are additionally complicated by two NPC assembly modes, postmitotic and interphase assembly. In this work, the authors address the mechanism of postmitotic NPC assembly in mammalian cells using an in vivo approach based on a precisely timed chemically induced depletion of NPC components.

Key results

Specifically, this work aims to probe the role of the central channel cohesive FG-repeat nucleoporin Nup62 in postmitotic NPC assembly. To this end, the authors combine state-of-the-art imaging techniques (electron tomography and high-resolution optical imaging using DNA-PAINT and MINFUX superresolution microscopy) with advanced chemical genetic approaches to controllably and quickly deplete Nup62. By these, the authors show that removal of Nup62 through induced proteasomal degradation just prior to postmitotic NPC assembly disrupts the Nup62-containing subcomplexes Nup62-Nup54-Nup58 in the central channel and Nup214-Nup88-Nup62 in the cytoplasmic face, leading to their assembly failure and partial degradation of these Nup62-interacting NUPs.

On the ultrastructural side, the authors show that this disruption leads to a failed NPC dilation, which is accompanied by irregular spacing between the inner and outer nuclear membranes and the lack of nuclear expansion. The authors show that the latter two defects can be clearly uncoupled from the NPC assembly defect by blocking nucleocytoplasmic transport at the moment of postmitotic nuclear envelope reassembly. These results are the basis of their two-step nuclear assembly model in which NPC assembly and, consequently, active nucleocytoplasmic transport are prerequisites for nuclear expansion and nuclear membrane flattening.

A surprising result of this work is that relatively low concentrations of 1,6-hexanediol, 1.5% - a compound known to disrupt cohesive FG repeat interactions - mimic the effect of Nup62 depletion without disrupting the association of Nup62 with postmitotic NPC. All in all, this led the authors to conclude that the cohesive FG repeat interactions are a key factor in postmitotic NPC assembly. Their condensation in the central channel produces outward-pushing force that causes the NPC to dilate. In support, they provide molecular dynamic simulations of condensates formed by FG repats in Nup62-Nup54-Nup58 and Nup214-Nup88-Nup62 complexes, specifically showing that the former can produce condensates with biophysical properties (surface tension is used as a metric) capable of membrane bending.

We sincerely thank the reviewer for this clear and thoughtful summary of our work. We greatly appreciate the careful synthesis of both the conceptual motivation and the mechanistic conclusions of the study.

Validity

1. In Figure 2 and Supplements 1- 4 the authors rigorously investigate the effect of nup62 depletion on the localization of the immediate Nup62 interactors, Nup88, Nup214, Nup58, and Nup54, as well as the more distant NUPs Nup133, Nup98 Nup155, Elys , Nup153 and Tpr. Except

for the immediate interactors (Nup88, Nup214, Nup58, and Nup54) and Tpr, they observe that the NUPs generally normally localize to the postmitotic nuclear envelope. Based on these findings, they conclude that the defect in the NPC assembly is due to selective disruption of two Nup62-containing complexes (Nup62-Nup54-Nup58 and Nup214-Nup88-Nup62) and the lack of their NPC incorporation. Considering the reduced NPC diameter observed by the authors, it is curious what is happening with Nup93? Does it still localize to NPCs upon Nup62 depletion? Together with the Nup62-Nup54-Nup58 complex, Nup93 makes up the inner ring of the NPC and, intuitively, can account for the establishment of the proper NPC diameter. Its yeast ortholog Nic96 forms a coiled coil bundle with Nsp1-Nup49-Nup57 heterotrimer and acts as assembly sensor, as was shown by the Hoelz group (DOI: 10.1126/science.aac9176). Potentially, the lack of Nup93 incorporation could contribute to the NPC dilation defect.

We thank the reviewer for this important and mechanistically insightful question. Given the central role of Nup93 within the inner ring scaffold, we agree that its recruitment status is potentially important for fully understanding the dilation defect of Nup62 depletion.

To directly address this point, we performed live-cell imaging experiments by transiently expressing fluorescently tagged Nup93 in our Nup62-mEGFP-FKBP^{F36V} cell line. Upon acute Nup62 depletion prior to postmitotic assembly, we did not observe defects in Nup93 recruitment to the reforming nuclear envelope. Nup93 displayed a localization pattern indistinguishable from control conditions.

These results indicate that the reduced pore diameter is unlikely to arise from defective incorporation of the inner ring scaffold. Instead, they support our conclusion that scaffold assembly, including Nup93 recruitment, proceeds normally upon acute Nup62 depletion, whereas Nups directly or functionally associate with the Nup62 are selectively affected. We have now included the data in our revised Fig. 2F and Extended Data Fig. 7F, and revised the manuscript accordingly (line 165).

2. In Figures 3A-C and Figure 3 supplement 1 authors quantitatively model the condensation propensity of Nup62-58-54 complexes (based on a pre-trained MD model) and conclude that in the confined environment of the NPC these FG repeats would form a condensate whose surface tension overcomes the threshold for membrane bending. This is speculated to generate an outward pushing force to "open" the NPC. Can it be excluded that these forces lead to a collapse rather than dilation and that it is some other mode of FG-repeat interactions that drives the dilation process? Cohesive FG repeats also interact with NTRs and the Roderick Lims group showed multiple evidence that NTRs, though their FG interactions, form an integral part of the NPC and contribute to the integrity of the nucleocytoplasmic barrier (for example, 10.1083/jcb.202108107). Can these interactions contribute to NPC dilation? In has also been reported that cohesive FG repeats bind to scaffold NUPs (10.1016/j.cell.2017.09.033) and contribute to association of cohesive FG-NUPs with the NPC and to NPC assembly. Can it be that these Velcro-type interactions make a dilated NPC a more energy-favorable state?

We thank the reviewer for the thoughtful and mechanistically insightful questions. We agree that it is important to clarify why condensation of the Nup62-58-54 FG network is expected to bias the system toward dilation rather than collapse, and to acknowledge that additional FG-mediated interactions may also contribute to pore dilation.

To address the first point, we performed an additional simulation comparing the conformational size of a single Nup62-58-54 subcomplex in isolation with that of the same subcomplex within a

condensate. Consistent with Flory-Huggins solution theory (DOI: 10.1063/1.1723621), the Nup62-58-54 subcomplex expanded upon condensation due to a more favorable solvent environment within the hydrophobic condensate phase. This increase in the volume occupied per subcomplex provides a direct physical rationale for why condensation is expected to exert an outward mechanical bias to its surroundings, making pore dilation more likely than collapse. We have included the new data in the revised Extended Data Fig. 10D and E and revised in the Results accordingly (line 228-233).

We also agree that cohesive FG-repeat condensation is unlikely to be the only interaction relevant during postmitotic NPC assembly. We therefore expanded the Discussion (line 403-407) to explicitly note that additional interactions, including additional FG-Nups through entropic exclusion, FG-NTR interactions and FG-scaffold interactions, may contribute to pore expansion and stabilization. Thus, while our data support a central role for the Nup62-58-54 FG network in driving dilation, they do not exclude cooperative contributions from transport factors or scaffold binding during NPC maturation.

3. The authors strengthen their argument about the role of cohesive FG-repeat interactions by showing that low concentrations of 1,6-hexanediol (HDX) mimic the in vivo effect of Nup62 depletion without disrupting nup62 localization (Figure 3D-H and Supplementary 2-4). It is curious whether MD simulations can recapitulate the hexanediol effect, i.e. to reduce the surface tension below the membrane-bending limit? As HDX effects on FG condensation can be reverted (10.1016/j.cell.2007.01.044), it is also interesting whether this applies to NPC assembly.

We thank the reviewer for this insightful suggestion.

To directly test whether MD simulations could recapitulate the effect of 1,6-hexanediol (HXD) on FG-Nup condensation, we extended our simulations to explicitly incorporate the impact of HXD on FG-Nup interactions.

Based on Flory-Huggins theory (DOI: 10.1063/1.1723621) and previous studies of intrinsically disordered proteins, we reasoned that the monomeric conformational properties of FG-Nups are predictive of their phase-separation propensity. We therefore performed all-atom simulations of a representative FG-Nup segment in the presence of 0%, 1.5%, and 5% HXD. These simulations allowed us to refit our coarse-grained model interaction strength (ε, Eq. 1 in the Methods section) to match the experimental results, creating a new model for 1.5% and 5.0% HXD. The process is described in more detail in the revised Methods section ("Modifying MOFF to account for hexanediol treatment").

Using this revised model, we then evaluated the phase-separation propensity of the Nup62-54-58 subcomplex. Increasing HXD concentration progressively reduced condensation, as reflected by lower protein concentrations in the dense phase and higher concentrations in the dilute phase (revised Extended Data Fig. 11D-F). Notably, at 1.5% HXD the predicted saturation concentration (~10 μM) exceeds the estimated cellular concentration of Nup62 (~0.2 μM) (DOI: 10.1038/s41586-022-05528-w), indicating that this dosage would suppress phase separation under physiological conditions. These simulations therefore support our experimental observation that low-dose HXD disrupts hydrophobic interactions within Nup62-58-54 subcomplexes in vivo.

We have incorporated these analyses into the revised figure (Extended Data Fig. 11D-F) and updated the manuscript text accordingly (line 239-246).

To test whether the effects of HXD could be reversed, we performed simulations in which we started from the configuration produced at the end of the simulation with HXD and then restored the 0% HXD interaction parameters. In these simulations, high-density condensates similar to those seen with 0% HXD are rapidly recovered (within 5 μs), although multiple condensates are observed due to the slow timeframe of droplet fusion relative to droplet formation (see the figure below). We note that this simulation result was expected, as all simulations are performed at equilibrium. For completeness, we provide these simulation results here for transparency.

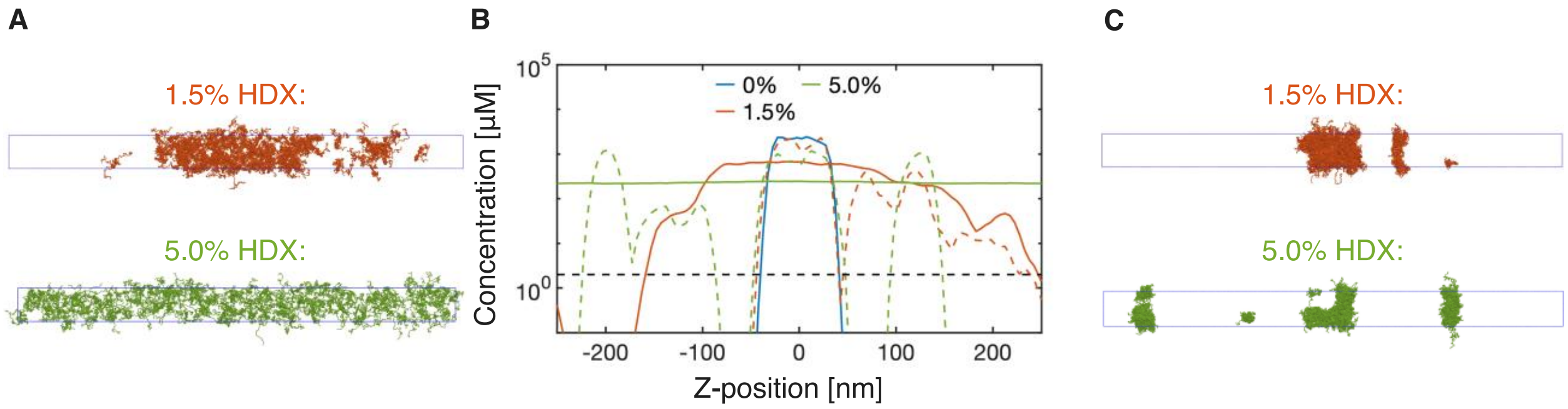

Figure Legend: Testing simulation ability to recover phase separation after HXD-treatment. A) Images of the final frame from the simulation from simulations with HXD. B) Slab density profile on a log scale as a function of box length for the full length Nup62-58-54 subcomplex with specified amounts of HXD. Dashed lines indicate simulations in which HXD has been removed. C) Images of the final frame from the simulation from simulations in which HXD has been removed.

Significance

The ultimate strength of this study is the establishment of a controllable system to quickly remove select NUPs (or even other proteins) exactly prior to mitotic exit, thus allowing a systematic analysis of the postmitotic NPC assembly mechanism and avoiding the danger of indirect effects. Perhaps such a chemogenetic strategy can also be applied to other cellular processes/factors beyond NUPs.

Although the direct role of cohesive FG interactions has yet to be more firmly established, this work contributes to our understanding of the NPC assembly showing in vivo a specific role of Nup62 in the NPC dilation step and causally linking the NPC dilation with the establishment of the nucleocytoplasmic transport function and nuclear expansion/nuclear membrane flattening. It is also highly unexpected that HDX can inhibit NPC dilation at such low concentrations. In the future, this finding may shed light on the mechanism of NPC assembly.

The work also highlights the utility of various chemogenetic strategies (the FKBP12F36V – dTAG system and LAMA) and how they can be used to unravel complex cellular processes.

We sincerely thank the reviewer for this generous evaluation of our work. We are pleased that the reviewer highlights the strength of the temporally controlled chemogenetic strategy and its potential applicability to other dynamic cellular processes beyond NPC assembly.

Data and methodology

The problem of postmitotic NPC assembly is addressed with adequate experimental techniques - well-characterized Nup62 depletion combined with a correlation microscopy analysis on precisely timed single cells following the anaphase onset. The authors also used an elegant way

to disrupt the directional nucleocytoplasmic transport by a chemogenetically controlled release of RanT24N from mitochondrial membrane sequestration (Figure 4 and Supplements).

Although the role of cohesive FG interactions is probed with the best tools available, i.e. HDX, the mechanistic connection between cohesive FG-FG interactions and NPC dilation might be hard to draw solely from this evidence, as HDX can potentially inhibit other FG-interaction modes and even other types of binding between the NPC components.

We thank the reviewer for this thoughtful and balanced assessment.

We agree that hexanediol is not a perfectly specific perturbant and may affect interactions beyond cohesive FG-FG contacts. For this reason, we interpret the temporally controlled HXD experiment together with several independent lines of evidence. These include: (i) selective depletion of the Nup62-58-54 complex, which arrests NPC maturation specifically at the dilation step; (ii) molecular simulations indicating that condensation of the Nup62-58-54 complex can generate an outward mechanical bias consistent with pore dilation; (iii) preservation of central FG repeats together with intact outer and inner ring scaffold assembly, which is nevertheless insufficient to drive pore dilation in the absence of functional cohesive FG interactions; and (iv) perturbation of nuclear import demonstrating that import-driven membrane tension is not responsible for pore dilation. These complementary approaches converge on a model in which cohesive interactions within the Nup62-containing FG network play a key role in driving pore dilation during postmitotic NPC assembly.

We agree that more targeted perturbations of FG interactions would further strengthen this mechanistic link. However, developing such an experimental approach that allows acute perturbation during NPC assembly would require substantial additional development and are beyond the scope of the present revision. We have therefore noted this limitation in the revised Discussion (line 400-402) and highlight it as an interesting direction for future work.

Analytical approach

The experimental data presented in the manuscript are generally of high quality, both from the perspective of using appropriate controls and from the adequate statistical analysis of the experiments. The chemogenetic tools used for Nup62 depletion (the FKBP12F36V – dTAG system) and for controlled release of RanT24N (using the LAMA system) are thoroughly characterized. However, aside of basic "sanity checks" (see suggested improvements) this reviewer does not have adequate expertise to judge the MD aspect of this work.

We thank the reviewer for the careful and constructive evaluation of the experimental aspects of our study. We also appreciate the reviewer's transparency regarding the MD component.

Suggested improvements

Testing whether Nup93 still localizes to the NPC upon Nup62 depletion would be helpful to strengthen the argument that the Nup62-Nup54-Nup58 complex is the key factor in postmitotic NPC dilation (see validity).

We thank the reviewer for this helpful suggestion. This point has been addressed in our response to Validity – point 1, where we show that Nup93 recruitment to the reforming nuclear envelope remains unaffected upon Nup62 depletion.

It might be too much to ask, but if it is possible to pretrain the MD model in the presence of HDX it would be great to test how strongly 1.5% HDX reduces the condensate surface tension/prevents Nup62-54-58 condensate formation and if this effect can be reverted by HDX removal.

It would be great to test whether the HDX effect on the NPC assembly is reversible, i.e., whether HDX washout resolves the dilation defect as it happen in cease of cytosolic FG condensates (see validity).

We thank the reviewer for this helpful suggestion. This point has been addressed in our response to Validity – point 3, where we incorporate HXD into the MD simulations and discuss the reversibility of the HXD effect.

Regarding testing the HXD reversibility experimentally, we agree that testing whether the HXD-induced dilation defect can be reversed would be informative. However, because NPC dilation occurs within a narrow temporal window immediately after mitotic exit and structural analysis relies on high-resolution ultrastructural readouts at defined time points achieved by an advanced correlative workflow, it is technically very difficult to precisely align HXD washout with functional recovery of the FG network and the corresponding correlative structural readout. As such, this experiment is very challenging to implement within the scope of the present revision.

As there is no guarantee that HDX is a highly specific inhibitor of FG-FG interactions, the authors shall consider toning down stating their role as the driving force of NPC dilation, discussing the possible role of alternative FG interaction modes, and explaining in more detail how mechanistically the surface tension of the FG condensates can lead to NPC dilation.

We thank the reviewer for this important and thoughtful comment. We agree that HXD is not a perfectly specific perturbant and that mechanistic conclusions should not rely on HXD alone. Accordingly, we have moderated the wording in the revised manuscript to avoid implying that cohesive FG-FG interactions are the sole driving force of pore dilation. Instead, we now state that our data support a central contribution of the Nup62-58-54 FG network to the dilation step while allowing for additional FG-mediated interactions.

To further clarify the mechanistic basis of our model, we expanded our simulations to test whether condensation would bias the system toward collapse or dilation. We compared the conformational size of a Nup62-58-54 subcomplex in isolation with that of the same subcomplex within a condensate. The results suggest that the subcomplex expands upon phase separation due to a more favorable solvent environment in the condensate. This increase in per-molecule volume provides a physical rationale for how condensate surface tension could exert an outward mechanical bias on the surrounding scaffold, thereby favoring pore dilation (see also our response to Validity – point 2).

In addition, we expanded the Discussion to explicitly consider potentially cooperative interaction modes, including contributions from additional FG-Nups through entropic exclusion, interactions between FG repeats and nuclear transport receptors, and interactions between FG repeats and scaffold nucleoporins. We now state explicitly that these mechanisms may cooperate with the Nup62-containing FG network to promote or stabilize the dilated pore state during assembly (see also our response to Validity – point 2).

Clarity and context

The text and figures of the manuscript are well organized and easy to follow. Minor comment: in Figure 1_Supplementary 1 C-F it is not clear how the decay curves were obtained.

We thank the reviewer for this helpful comment.

The decay curves were obtained by fitting the kinetic data using a global one-phase exponential decay model with a free plateau, treating each replicate measurement as an individual data point. The fitting was performed using GraphPad Prism, and we have clarified this in the revised figure legend for Extended Data Fig. 1C-F (line 1447-1449).

References

Authors should check the integrity of the citations more carefully in discussion section. For example, in lines 327-328 the citations [56,57] do not seem to match the statement they support.

We thank the reviewer for pointing this out.

We have carefully re-examined the citations throughout the Discussion section and corrected the references to ensure that they accurately support the corresponding statement. In addition, we performed a comprehensive cross-check of all citations in the manuscript to prevent similar inconsistencies.

Expertise note

The interpretation of molecular dynamic simulations of FG-repeat interactions is outride of this reviewer's expertise.

We thank the reviewer for the transparency regarding the MD component.

Reviewer #3 (Remarks to the Author):

My main concern is the inaccurate method used to calculate surface tension in the molecular simulations, which play a central role in the researchers' main hypothesis.

The authors write, 'Molecular dynamics simulations predicted that 32 copies of the central channel subcomplex can self-associate via hydrophobic interactions to occupy the volume required for full pore size and exert an outward pushing force when recruited into the previously unoccupied pore centre.' Therefore, the simulations performed and the measurement of forces (surface tensions) play an essential role in the current hypothesis. The authors analysed the surface of the formed protein liquid droplets according to Henderson and Lekner's theory, which has previously been used to calculate the surface tension of FUS droplets from molecular dynamics simulations. The main idea is to calculate the surface tension from the spherical space anisotropy due to thermal fluctuations. However, this requires the shape of the droplet to be dominated by actual collective effects with all shape fluctuation modes being accessible. The droplet shown in Fig. 3 has evidently far to little constituents (32!) to adopt a shape whose fluctuations justify such thermal averaging. In fact, the non-smooth droplet shape seems completely dominated by the presence of too little constituents. Consequently, the surface tension measured via this route is highly inaccurate. Fortunately, the estimation can be substantially improved by a much simpler, more accurate method that exploits the implicit solvent nature of these simulations (in contrast, Hummer performed these simulations in explicit solvent!). The authors should do the following:

The MOFF force field is an implicit solvent force field. As there is no hydrostatic pressure, the virial pressure measured within the simulation stems solely from the interactions between the proteins (all simulation packages output box pressures). According to the virial theorem, pressure is given by: P = 2/V_(box) (E_(kin) – Virial). To obtain the surface tension via this more robust mechanical route, the authors should extract the isotropic box pressures measured in the simulations performed at a constant box volume. As the box volume is largely void, these values will be very small. They should then rescale the isotropic pressure value by multiplying it by the factor V_(droplet)/V_(box), where V_(droplet) is the volume of the droplet (already estimated by the authors in their work) and V_(box) is the chosen volume of the simulation box. This rescaling effectively compensates for the arbitrary box size. In the case of a solvent-free model only, the pressure calculated via this method is the so-called Laplace pressure (ΔP), as the hydrostatic pressure is 0 by definition in a solvent-free model and therefore P_(simulation) equals ΔP. The surface tension can then be obtained using the formula ΔP = (2σ)/R, where R is the average radius of the droplet (assume its spherical and resolve the effective radius from the known droplet volume) and σ is the surface tension of interest.

Reviewer #3 (Remarks on code availability):

n.a

Reviewer #4 (Remarks to the Author):

I have co-reviewed this manuscript with reviewer #3.

We thank the reviewers for their careful evaluation of our molecular dynamics analysis and for suggesting a more robust approach to estimate condensate surface tension, which we have evaluated and indeed found to be superior and therefore adopted in our revisions.

Following the suggestion, we calculated the surface tension using the simulation pressure instead of the shape of the condensate. The revised text (line 932-943) reflects the new method used to calculate surface tension:

> Simulations were first analyzed to determine the radius and surface tension of the droplet. Surface tensions (τ) were calculated according to the Young-Laplace equation
>
> $$\tau = \frac{R\Delta P}{2}, \quad (3)$$
>
> where R is the radius of the droplet and $\Delta P = P_{in} - P_{out}$ is the change in pressure from inside the droplet (P_{in}) to outside the droplet (P_{out}). Since our simulations were in implicit solvent, P_{out} was 0. The internal pressure of the droplet could be calculated from the virial pressure of the simulation box (P_{box}) by
>
> $$P_{\text{in}} = \frac{V_{\text{box}}}{V_{\text{drop}}} P_{\text{box}}, \quad (4)$$
>
> where V_{drop} is the droplet volume and V_{box} is the volume of the simulation box. V_{drop} was calculated by assuming the droplet was a sphere of radius R. R was determined by fitting the radial mass density around the largest cluster, c(r), to a step function of the form
>
> $$c(r) = B - A\text{erf}\left(\frac{r-R}{\sqrt{2}W}\right), \quad (5)$$
>
> where r is the distance from the droplet center of mass, erf is the error function, and A, B, R, and W are determined by fitting.

Originally, pressure was not tracked during the simulation, so all simulations were rerun to be able to calculate surface tension. Analyses outside of surface tension (condensate volume, contact maps, condensate organization, R_g) did not qualitatively change upon rerunning the simulations, supporting their convergence.

However, using pressure instead of condensate shape does cause significant differences in the condensate surface tension (previous method: 2.2 nN/µm, 95% CI: [1.5 nN/µm, 2.8 nN/µm]), (new method: $3.3*10^{-3}$ nN/µm, 95% CI: [$1.5*10^{-3}$ nN/µm, $5.1*10^{-3}$ nN/µm]). As noted by the reviewer, our initial approach relied on deviations of the condensate shape from a spherical geometry to calculate surface tension. For very small condensates, this method likely overestimated surface tension due to the rigidity of the ordered domains, which cause deviations from spherical geometries not due to condensate surface tension.

The new surface tension values estimated in using the pressure instead of shape also align better with experimental measurements of condensate surface tensions (typically 10^{-1}-10^{-4} nN/µm) [Gouveia, B et al. Nature 2022, 609, 255]. Although significantly smaller, the new estimate of surface tension remains consistent with our general hypothesis that condensate surface tension can cause membrane bending. For example, FUS forms condensates with surface tensions

estimated to be in a similarly low range ~10^{-2} nN/µm [Benayad, Z. et al. J. Chem. Theory Comput. 2021, 17, 525] and can induce significant membrane reformation when targeted to a membrane [Yuan, F. et al. PNAS, 2021, 18, 11]. We now note this explicitly in the discussion (line 443-445), with corresponding citations included in the revised text:

> "Molecular dynamics simulations furthermore suggest that the surface tension of individual FG-Nup condensates (~$3.3*10^{-3}$ nN/µm) is similar to FUS condensates (~10^{-2} nN/µm), which have shown an ability to remodel membranes in vitro."

We have entirely removed the comparison of condensate surface tension with membrane tension as this is no longer appropriate with the revised pressure-based calculation. In addition, our experimental data that demonstrate that acutely inhibiting nuclear import, leads to decrease of nuclear membrane tension but does not impair pore dilation, indicates that membrane tension is unlikely to limit this step of postmitotic NPC assembly.

Overall, we therefore propose that localized FG-Nup condensate formation might generate localized capillary pressure that acts within the confined geometry of the forming nuclear pore. This revised interpretation is consistent with the magnitude of the pressure-based surface tension estimate, and with the timing of postmitotic NPC assembly prior to the establishment of import-driven nuclear membrane tension.

We would like to thank the reviewers again for suggesting a more robust tension estimation method. We believe that adopting this approach in our revision strengthen the physical basis of our model and allowed us to be more precise regarding our proposed model that hydrophobic interactions of FG-Nups contribute to pore dilation during postmitotic NPC assembly.

Reviewer #5 (Remarks to the Author):

The manuscript entitled "Hydrophobic interactions of FG-nucleoporins are required for dilating nuclear membrane pores into selective transport channels after mitosis" addresses a compelling research question in structural biology: how NPCs reform and reestablish their functional architecture during mitotic exit. Specifically, it investigates the role of the largely intrinsically disordered central channel subcomplex Nup62–58–54 in this process. The NPC is undoubtedly one of the most fascinating macromolecular assemblies, and the authors focus in this paper on the central transport channel and its intrinsically disordered FG-domains. This region is particularly challenging to characterize structurally due to its inherent disorder, yet it is indispensable for establishing the NPC's selective permeability barrier.

Overall, I find the study to be of high quality. The topic is highly interesting for NSMB and a broad scientific readership. Nevertheless, I believe the manuscript requires additional validation in some of its key results before it can meet the standard expected for publication in NSMB. One major concern relates to one of the primary experimental readouts, which are the NPC diameter measurements. These were obtained using a classical TEM sample preparation workflow including high-pressure freezing, freeze substitution, resin embedding, ultrathin sectioning, and heavy-metal staining. This approach is well known to introduce several artifacts, in contrast to the current gold standard in NPC structural research, namely cryo-FIB milling combined with cryo-ET. This raises concerns about the accuracy of the reported diameters. In addition, the MD simulations presented in the paper would benefit from closer adaptation to the native NPC geometry in order to ensure that the conclusions drawn from these analyses are robust.

In my view, the manuscript has strong potential to make a meaningful contribution to the field. Addressing the following major points would considerably strengthen the work and bring it closer to the standard expected for publication in NSMB.

We sincerely thank the reviewer for this thoughtful and balanced evaluation. We greatly appreciate the recognition of the conceptual importance of the work and its potential relevance to a broad readership.

We also take seriously the reviewer's concerns regarding (i) possible artifacts associated with freeze substitution-based TEM workflows and (ii) the geometric realism of the MD simulations. Below, we address each of the reviewer's specific points in detail.

Major points

In the NPC side-view images (e.g., Fig. 1B) and in the tomograms available to the reviewer, the ΔNup62 condition appears to show very intense staining within the central channel. This observation is counterintuitive, as the absence of Nup62 would be expected to result in reduced density in the central channel. What exactly is being stained in this condition? Could the strong accumulation of stain artificially bridge or "glue" the central channel, thereby preventing NPC dilation, not as a direct consequence of the loss of Nup62 but rather as an artifact of staining? This point requires clarification and should be addressed explicitly in the discussion.

We thank the reviewer for this careful observation and for raising this important point.

The electron density observed in the central region under ΔNup62 conditions most likely reflects staining of remaining structural elements of the assembled NPC prior to pore dilation, particularly components of the inner ring scaffold, which remain incorporated upon Nup62 depletion. Heavy-metal stains preferentially label structured proteinaceous material and membranes, and therefore

highlight the scaffold architecture that persists even when the central channel complex is absent. This interpretation is consistent with our compositional analyses (Fig. 2 and related supplementary figures), which show that inner ring components remain assembled despite depletion of Nup62.

Importantly, control and ΔNup62 samples were processed identically within the same high-pressure freezing and freeze-substitution batches, ensuring equivalent sample-preparation conditions across experimental groups. Any staining-related effects would therefore be expected to affect both conditions similarly. In addition, high-pressure freezing followed by low-temperature freeze substitution is widely used to preserve cellular ultrastructure close to the native state (DOI: 10.1002/jemt.1060130305; DOI: 10.1007/978-1-0716-2639-9_13; DOI: 10.1007/s00418-008-0500-1; DOI: 10.1111/jmi.12875). Any dimensional changes introduced during dehydration and resin embedding typically manifest as relatively uniform shrinkage (DOI: 10.56507/SCLL2742; DOI: 10.7554/eLife.19071; DOI: 10.1038/s41594-017-0001-9), rather than heterogeneous distortions between material-dense and material-poor regions at the subcellular level, making selective narrowing of the nuclear pore due to staining unlikely.

Further supporting this conclusion, the dilation defect is independently observed using correlative MINFLUX imaging (Fig. 1F and G), which relies on a completely different experimental principle and does not involve heavy-metal staining. The convergence of these orthogonal approaches strongly argues that the observed pore constriction reflects a bona fide structural phenotype rather than an artifact of sample preparation.

We have now clarified this point in the revised text (line 110-111 and line 116-118) to explicitly address the EM fixation method (high-pressure freezing) we used and the likely origin of the central density in the absence of Nup62.

Schuller et al. (Nature, 2021, “The cellular environment shapes the nuclear pore complex architecture”) demonstrated using cryo-FIB and cryo-ET that the diameter of native NPCs is considerably larger than previously expected. In their study, the diameter of HeLa NPCs was measured at 111 nm, substantially greater than the 89 nm reported by von Appen et al. (Nature, 2015, “In situ structural analysis of the human nuclear pore complex”) using cryo-ET and subtomogram averaging on isolated nuclear envelopes. Both of these values are markedly larger than the ~70 nm diameter reported in the present manuscript for the DMSO control condition (Fig. 1C). Notably, the cryo-FIB/cryo-ET measurements suggest a HeLa NPC diameter ~40 nm larger than that described here. From my perspective, it is highly problematic that this discrepancy is not addressed in the manuscript, and it should be explicitly discussed.

We thank the reviewer for raising this point.

The diameters reported in our study were obtained from high-pressure frozen, freeze-substituted, and resin-embedded samples. It is well established that these preparation procedures can introduce sample shrinkage, resulting in systematically smaller apparent dimensions compared to cryo-preserved specimens (DOI: 10.56507/SCLL2742; DOI: 10.7554/eLife.19071; DOI: 10.1038/s41594-017-0001-9). Previous work comparing cryo-ET with resin-based TEM has reported such discrepancies in nuclear and NPC measurements (DOI: 10.7554/eLife.19071; DOI: 10.1038/s41594-017-0001-9), and our absolute values fall within the range typically reported for resin-embedded samples (DOI: 10.7554/eLife.19071; DOI: 10.1038/s41594-017-0001-9; DOI: 10.1016/j.cell.2017.10.008; DOI: 10.1083/jcb.201706024).

Importantly, our study focuses on relative differences in pore diameter between conditions measured within the same preparation and imaging workflow, for which such systematic shrinkage effects are expected to apply uniformly across samples and therefore do not affect the comparative analysis. To avoid potential confusion arising from direct comparison of absolute values across different preparation methods, we have revised the relevant statements to address relative changes (line 137-139).

We have now explicitly addressed this discrepancy in the revised Methods section (line 795-800), citing prior studies that report preparation-dependent variation in NPC diameter and reporting the extent of specimen shrinkage relative to cryo-ET measurements in the same cell line.

Apart from the discrepancies in diameter measurements compared to cryo-methods, a key concern is the validity of the measured diameter differences between the experimental conditions, namely the DMSO control and the ΔNup62 condition (Fig. 1B, 1C), and the interphase control and the HXD condition (Fig. 3E, 3F). The profile-based diameter measurement procedure is susceptible to several potential sources of error. For example, how precisely can the center of an individual NPC be determined, particularly when pores are slightly tilted or deviate from a circular shape? How reliably can the membrane be distinguished from the spoke ring, especially under stain-based preparation? What is the effect of knife compression during sectioning, and to what extent do dehydration, resin polymerization, or sample pre-irradiation contribute to systematic errors in the diameter measurements?

Regarding pre-irradiation, the Methods section (line 733) reports an electron dose of 5-10 e^-/nm^2. On what basis is a sample irradiated at, for example, 10 e^-/nm^2, and should such a sample not exhibit smaller NPC diameters than one irradiated at 5 e^-/nm^2? To address these concerns, a comprehensive error analysis of the reported diameter measurements would be valuable. This should include a clear assessment of the measurement uncertainties (e.g., pore center detection, elliptical pores, pore tilt), systematic errors (e.g., knife compression, knife orientation, sample shrinkage), and the propagation of these errors. The pore diameters for each experimental condition could then be reported as $x_{measured}$ ± uncertainty derived from the error analysis. In principle, subtomogram averaging could elegantly address some of the measurement uncertainties, as it for example inherently accounts for pore center determination and tilt. However, systematic errors in the diameter measurements would still need to be carefully quantified.

We thank the reviewer for this thoughtful and detailed assessment of potential sources of uncertainty in the diameter measurements.

This membrane-profile approach provides a geometrically well-defined readout of pore diameter at the center with correction of pore orientations, and has been successfully used in our previous studies to quantify NPC membrane openings with high reliability (DOI: 10.1038/s41594-017-0001-9; DOI: 10.7554/eLife.19071). In brief, to ensure accurate diameter measurements at the pore center, each NPC was individually aligned in 3D to obtain its true cross-sectional view prior to analysis. Specifically, tomographic sub-volumes containing individual NPCs were manually aligned in IMOD, sequentially correcting rotations around the Z, X, and Y axes until the pore axis was perpendicular to the viewing plane. Alignment was iteratively refined until no vertical or lateral displacement of the pore center was observed when scrolling through the tomographic slices. This procedure ensures that measurements are performed on the true cross-section of the pore, thereby minimizing uncertainties arising from pore tilt, center determination, or deviations from circular geometry.

Following orientation correction, the nuclear envelope membrane profile defining the maximal pore membrane opening was traced directly in the tomographic slices. Notably, under our staining conditions, the membranes are clearly resolved and can be readily distinguished from the spoke ring, as illustrated in the representative tomographic images shown in the figures. The traced membrane contours were subsequently analyzed using a custom MATLAB script (DOI: 10.1038/s41594-017-0001-9), which determines the pore diameter by fitting the membrane profile corresponding to the largest membrane-defined pore opening at the correctly oriented cross-section.

To directly address the reviewer's concern regarding pre-irradiation, we note that this step is routinely applied prior to tilt-series acquisition to stabilize the section and reduce beam-induced deformation. We have removed the previously reported electron dose values, as these were not accurately quantified. In practice, the pre-irradiation step was applied to the specimen at low magnification for 20 – 40 min prior to tilt-series acquisition.

To directly test whether pre-irradiation induces measurable shrinkage, we performed dedicated control experiments in which sections were subjected to irradiation periods of either 20 minutes (low dose) or 40 minutes (high dose) prior to tilt-series acquisition, closely matching the conditions used during our tomogram acquisition. NPC diameters were quantified using membrane-profile measurements, and we observed no detectable difference in pore diameter between the two irradiation conditions (Fig A and B, see below). Importantly, we further validated these measurements using subtomogram averaging, which inherently accounts for pore center determination and pore tilt. Membrane-profile-based measurements at the averaged pore centers yielded consistent pore diameters under both irradiation conditions (Fig C and D, see below), in agreement with the membrane-profile measurements obtained from individual NPCs (Fig A and B, see below). Together, these results indicate that (i) the membrane-profile approach provides a robust and sufficiently accurate method for quantitative determination of pore diameter, and (ii) the irradiation step used in our practice does not introduce detectable shrinkage affecting pore size measurements. The corresponding control data are provided below for transparency.

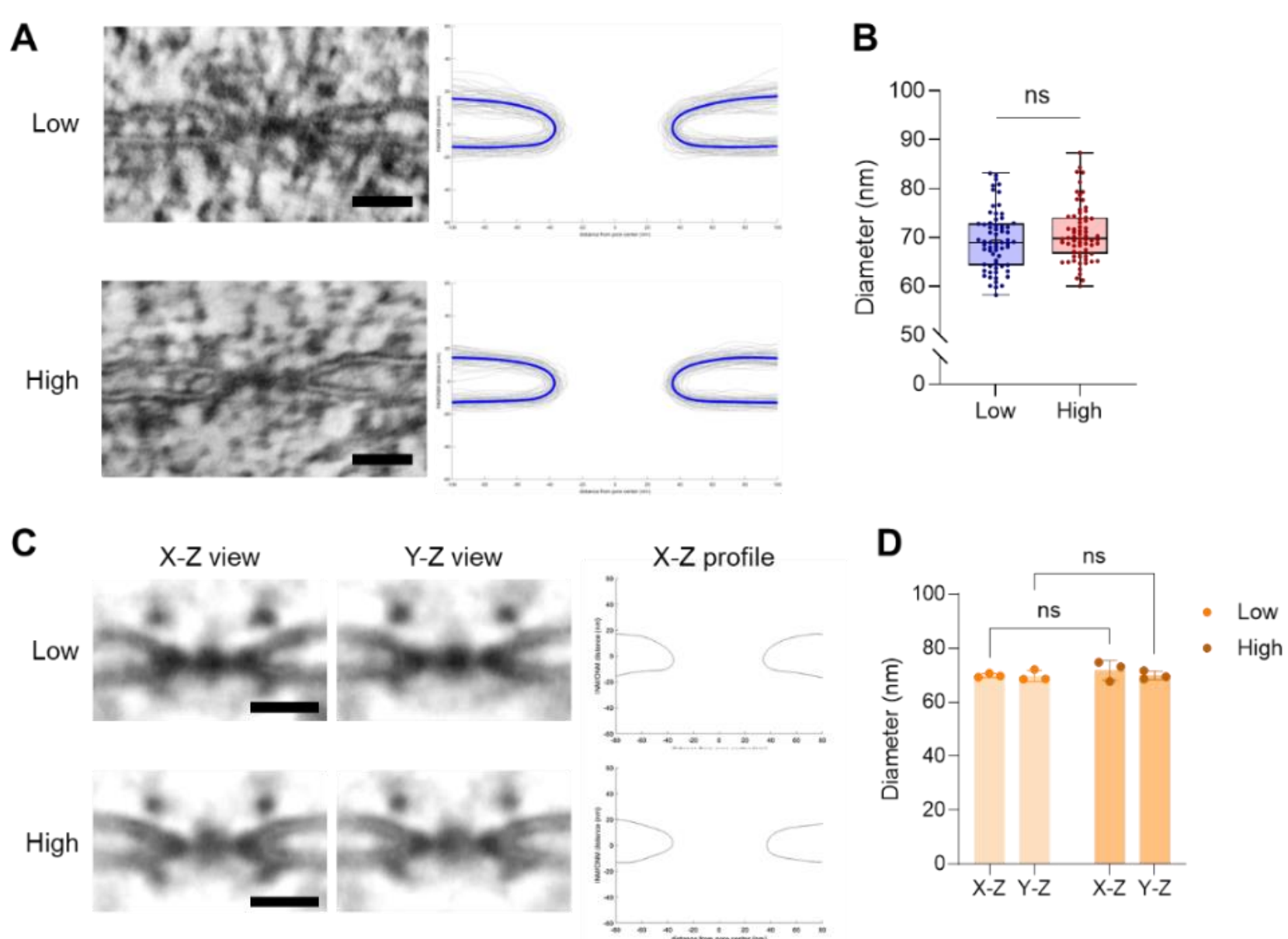

Figure Legend: (**A**) Left, tomographic slices showing cross-section views of nuclear pores assembled under low- (n=66) or high-dose (n=67) pre-irradiation. Right, membrane profiles of all measured pores, with mean profiles highlighted in bold. Scale bars, 50 nm. (**B**) Quantification of the NPC diameters based on the membrane profile measurement from (A). Box-and-whisker plot shows the median, range (min to max), and all individual data points. Welch's two-tailed t-test analysis indicates no significant difference (ns). (**C**) Left, electron tomographic slices (X-Z and Y-Z views) of averaged nuclear pores at the center derived from (A). Right, membrane profiles of X-Z views from the averaged pores. Scale bars, 50 nm. (**D**) Quantification of the NPC diameters at the pore center based on the membrane profile measurement from (C) (three replicates per condition and view). Bar plot shows the mean, standard deviation, and all individual data points. Two-way ANOVA analysis indicates no significant difference (ns) between conditions.

Regarding potential systematic artifacts introduced during sample preparation (e.g., knife compression and orientation during sectioning, dehydration, or resin polymerization), these effects would be expected to influence all samples processed within the same preparation batches in a similar manner. In our study, control and experimental samples were prepared in parallel, making it unlikely that such systematic effects could account for the condition-specific diameter differences observed between DMSO and ΔNup62 NPCs or between interphase and HXD-treated cells.

In response to the reviewer's comments, we have expanded the Methods section (line 781-786) to more explicitly describe the measurement pipeline and now include an assessment of measurement uncertainties (line 115), incorporating contributions from geometric alignment, membrane tracing, and potential systematic effects, as well as their propagation.

In its current form, the molecular dynamics (MD) simulation appears relatively inconclusive. One major issue is that the simulated condensate is poorly described in the manuscript. For instance, it is unclear where the α-helices of the Nup62-58-54 complex are located in the simulated condensate. Do they form an outer shell or ring around the condensate, as might be expected based on the native pore geometry, or is the simulated condensate instead a tangled cluster of α-helices and low-complexity FG domains? It could also be helpful in Fig. 3B to color the ordered and disordered domains in different colors and zoom into the assembly. How would the condensate behavior change if the linear order of residues in the FG domains were randomly permuted? Furthermore, the starting configuration of the simulation, a random placement of 32 copies of Nup62-58-54 in a box, completely neglects the cylindrical geometry of the central transport channel. From my perspective, the explanatory power of the MD simulations would be greatly improved if the starting configuration placed the 32 Nup62-58-54 copies on a ring, with their α-helices attached along the ring and the low-complexity FG domains protruding inward, modelling the more physiologically relevant arrangement in the NPC central channel. Moreover, also the comparison between the volume of the simulated condensate with the measured volume deficit (line 215-216) should include the measurement uncertainty of this quantities and discuss the large difference to the diameter values measured by cryo-FIB/cryo-ET methods.

We thank the reviewer for raising this important point.

The geometry of a spherical condensate was chosen intentionally in our modeling. Although the Nup62-58-54 subcomplex ultimately forms part of a ring-like arrangement within the mature pore, the structural state during early postmitotic assembly is not known. Our previous experimental and modeling work (DOI: 10.1038/s41586-022-05528-w; DOI: 10.1073/pnas.2415674122) suggests gradual incorporation of Nup62 subcomplexes, implying that a fully preformed inner ring

is unlikely to exist prior to central channel maturation. We therefore used an unbiased initial configuration (random placement of 32 subcomplexes) to allow spontaneous self-organization.

We agree that the simulations may offer a computational microscope that may allow for unique characterization of the condensate geometry. We reanalyzed our simulations to find which portions of sequence drive inter-subcomplex interactions, finding that the disordered, FG-rich segments primarily drive interactions (A, shown below). Further, we analyzed the organization of the condensate by comparing the distance from the center of the cluster between the ordered and disordered domains. From this analysis, we observed both “IDR-in” and “IDR-out” condensates (B, C shown below). The “IDR-in” condensates push the alpha-helical domains toward the exterior of the condensate, while the “IDR-out” condensates have alpha-helical domains closer to the condensate center. The “IDR-in” geometries we observe are suggestive of the configuration of the Nup62-58-54 subcomplex takes within the NPC – the helical domains remain on the exterior of the condensate and eventual pore, while disordered, FG-Nup segments localize near the center of the condensate and eventual pore. It is possible that these “IDR-in” conformations are a transition state before the ordered domains become anchored to the pore, completing the inner ring. However, computationally testing this hypothesis remains beyond the scope of this work. These results have been added to the revised manuscript (line 224-228), and the figures below are included as part of revised Extended Data Fig. 10A-C.

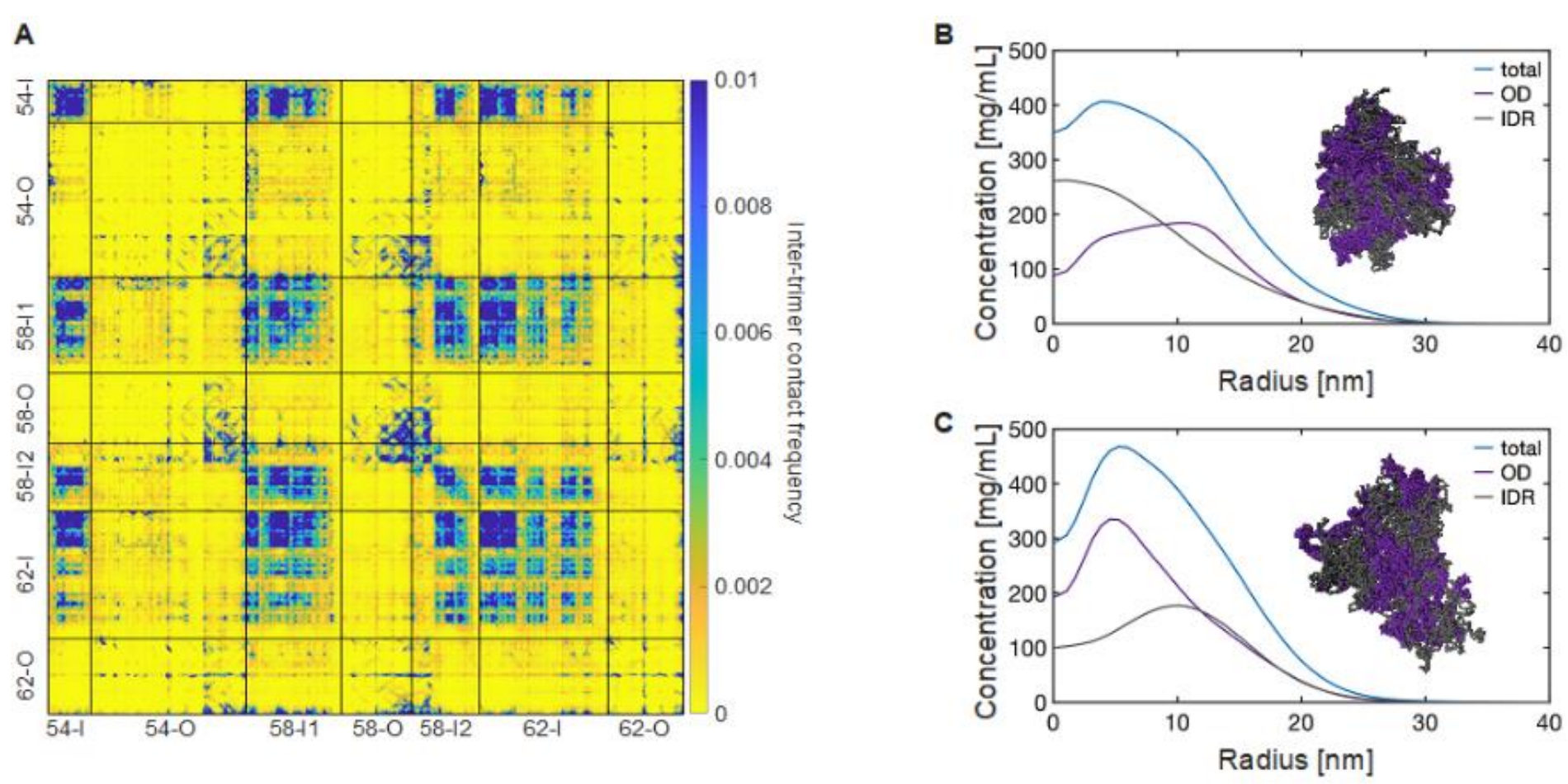

Figure Legend: Nup62-58-54 interactions colocalize disordered domains (A) Inter-subcomplex contact map of Nup62-58-54 subcomplex with color coding indicating contact frequency (axis labels: I – IDR, O – OD, # - Nup). (B, C) Plot of the average simulated OD and IDR concentration as a function of distance from the cluster center. Each plot is an average of 6 separate simulations, and condensate images are colored by residue (OD – purple, IDR - gray).

We have also incorporated error estimates into both the simulated condensate volume and the measured volume deficit, with the latter corrected for specimen shrinkage relative to cryo-ET measurements (line 218-222, line 953-957).

Reviewer #6 (Remarks to the Author):

As asked by the editor, I particularly had a look at the imaging and here again the Minflux part.

To start, I want to say that the correlative imaging done is impressive. This is a major achievement and must have been hard engineering work to make it work even at a leading imaging facility. The manuscript encompasses a multitude of imaging techniques that all must be executed well in combination with modeling. In that light my comments on the imaging/Minflux are smaller detail questions. Overall the LM imaging is done well and there only some questions/clarifications that I want to raise.

We thank the reviewer for this generous evaluation of our correlative imaging strategy and for recognizing the technical integration of advanced imaging modalities with quantitative modeling.

We are pleased that the overall light microscopy experiments are viewed positively and address the specific MINFLUX-related questions below.

It is unclear to me why 3D minflux is used, would not standard 3D PAINT/STORM work just as well? The required resolution to draw conclusions would not be compromised.

We thank the reviewer for this important methodological question.

While standard 3D SMLM approaches such as dSTORM or DNA-PAINT can achieve ~10-15 nm localization precision under optimized conditions, reaching consistent sub-10 nm precision in 3D typically requires specialized implementations (e.g., 4Pi STORM), which impose strict constraints on sample mounting and are incompatible with our correlative workflow using gridded live-cell dishes.

Although DNA-PAINT can achieve sub-10 nm precision under TIRF illumination, our structures of interest were located several micrometers above the coverslip. This required an imaging modality capable of deeper penetration while effectively suppressing out-of-focus background.

RESI-based strategies could in principle approach similar resolution; however, they require multiple rounds of washout and relabeling. Because our experiments were performed shortly after mitotic exit in semi-adherent cells, repeated wash steps posed a substantial risk of cell loss or structural perturbation. Indeed, recent work (DOI: 10.1101/2025.09.03.673830, Figure 4) has demonstrated nanometer-scale nuclear envelope alterations following even a single washout during 3D-MINFLUX imaging of nuclear pores. We therefore considered such iterative labeling strategies unsuitable for our system.

Under these experimental constraints, 3D-MINFLUX provides superior performance compared to conventional 3D SMLM approaches in our setup, without the need for iterative labeling or restrictive sample geometries, thereby enabling reliable structural interpretation. We therefore consider it the most appropriate approach for the biological question addressed here.

The authors use 3D minflux but never show a 3D image. There are only 2D images. Why is that? Fig 1F shows the distance of the pore from the focal plane, but not the 3D structure.

We thank the reviewer for this helpful comment.

The data shown in Fig. 1F were acquired using 3D MINFLUX, and the axial (Z) position of each localization was determined with nanometer precision and encoded by depth-based color coding. Thus, the datasets intrinsically contain three-dimensional spatial information. For visual

comparison of pore diameters between conditions, we selected locally flat membrane regions to minimize curvature effects and displayed the data in the figure.

We agree that this representation does not fully convey the 3D organization of the pores. To address this, we have now included 3D-rendered Y-Z views in the revised Fig. 1F and Fig. 4C, which explicitly visualize the axial arrangement of the structures and more clearly reflect the 3D nature of the data. In addition, individual pores with minimal tilt are now shown in both X-Y and X-Z views in the revised Extended Data Fig. 4A and Extended Data Fig. 15A, further illustrating the 3D organization at the single-pore level.

From the text it is not clear if 3D information is ever really needed/used or the 3D imaging is only used to be able to get a NPC in a non-flat membrane imaged well.

We thank the reviewer for this clarification request.

The use of 3D MINFLUX information was essential for quantitative analysis and not limited to improving imaging of NPCs in curved membrane regions. Following mitosis, the reformed nuclear envelope contains pronounced folds and is frequently tilted relative to the optical axis. Without axial (Z) localization, such tilt would distort lateral distance measurements and lead to biased diameter estimation.

3D coordinates were therefore required to correctly retrieve and register individual NPCs prior to analysis. First, 3D information is used for the clustering step that allows us to identify the ring-like NPCs. Subsequently, registering NPCs in 3D accounts for their various orientations and projecting each NPC onto the plane orthogonal to the least axis of the globally registered NPCs allows extracting geometric parameters independent of membrane curvature. This ensured that diameter measurements reflect true pore geometry rather than imaging angle. Detailed information regarding 3D segmentation for single-pore retrieval, registration and projection is available in the publicly accessible code repository (https://git.embl.org/heriche/minflux-npc.git).

We have further revised the Methods section ("MINFLUX data analysis") to explicitly describe the analytical use of 3D localization in our workflow.

Maybe I did miss it, but how is the 3D structure of the NPC actually image and used? As with 3D minflux you will see both sides of the NPC (or is only one side formed and labelled?), is this information projected away as only the radius is used in the end? In case this information is present, it would be good to use later e.g. figure 3.

If there is 3D information, please also show a 3D image in projection of the different views to be able to judge the image quality.

We thank the reviewer for this important and thoughtful comment.

In our 3D MINFLUX recordings, the complete 3D localization cloud of the labeled nucleoporin ELYS was acquired. ELYS is a nuclear outer ring component that localizes specifically to the nuclear side of the NPC, as demonstrated in our previous work (DOI: 10.1091/mbc.E20-11-0728). Thus, the detected localizations originate from the nuclear side of the pore rather than from both faces of the NPC.

The axial (Z) coordinates are not discarded during analysis. As now clarified in the revised Methods section ("MINFLUX data analysis"), 3D localization is integral to our workflow, enabling

identification and alignment of individual NPCs, followed by projection for accurate extraction of geometric parameters such as diameter (see also our response above).

As noted above, to better illustrate the three-dimensional nature of the data, we now include Y-Z views in the revised Fig. 1F and Fig. 4C, as well as X-Y and X-Z views of individual pores with minimal tilt in the revised Extended Data Fig. 4A and Extended Data Fig. 15A.

uncertainty estimation of the radius. The pore radius is the most important quantity in the manuscript. There is no effort done for an error analysis here. The values are solely based on the fitted 2D radius. From the distribution of the values p-scores are computed, but certain an individual measurement is, is kept in the dark here. This could be done more in-depth. This could include with the localization precision, linkage error, projection alignment, fitting error (see also below). All of that does matter little if the differences between conditions are large compared to the diameter or if all errors are unbiased and you acquire large numbers. However, there is no discussion on this point and 5% differences in the mean might get difficult to make hard.

We thank the reviewer for raising this important point.

In our analysis, pore diameters are derived from populations of individually measured NPCs, and all statistical comparisons are performed on these empirical distributions. The observed variance of these distributions therefore inherently reflects the combined contribution of multiple sources of uncertainty, including localization precision, fitting residuals, registration variability, and upstream experimental factors. As such, the reported variability provides a direct and conservative measure of the effective uncertainty associated with the measurement.

We have revised the manuscript (line 131-133) to make this point explicit and now report 95% CI for all mean diameter measurements, providing a direct estimate of the uncertainty associated with the population mean.

For transparency, all individual measurements, together with the number of analyzed pores per condition (see also Fig. 1G and Fig. 4D), are provided in the publicly accessible code repository (https://git.embl.org/heriche/minflux-npc.git).

Details:

** Line 128/129 the average diameters are given without uncertainties. Is this below 1 nm? In any case it would be nice to state them here as this will indicate how significant the different of 126 and 133 is and the 7 vs 15 nm mentioned later in the same paragraph. From Figure 1 I would expect that this is the center of the Gaussian fit? In this case the FWHM or std could be a measure for the uncertainty to zero order.*

We thank the reviewer for this helpful suggestion.

We agree that reporting uncertainties alongside the mean diameters improves interpretability. In the revised manuscript (line 131-133), we now report these mean diameters together with an uncertainty metric (95% CI of the mean).

** l628 Why is the the mean fluorescence between nucleus and cytoplasm calculated from the maximum along the optical axis? Or is the maximum projection used for segmentation? This part is a bit unclear.*

We thank the reviewer for this important clarification.

Maximum-intensity projections of the full z-stack were used for both segmentation and fluorescence quantification. The fluorescence signal in our experiments is spatially homogeneous within the segmented compartments, and all samples were acquired under identical imaging conditions without signal saturation. Under these conditions, this approach ensures that fluorescence from the entire nuclear and cytoplasmic volume is represented, independent of focal plane selection. Moreover, because the analysis is based on relative nuclear-to-cytoplasmic (N/C) intensity ratios, any systematic influence of the projection method would affect all conditions equally and therefore does not alter the comparative interpretation.

We have clarified this explicitly in the Methods section ("Quantification of correlative immunofluorescence", line 660).

L643 and following:

- The labelling with antibodies will introduce a linkage error. This is substantial compared to the localization precision. It would be good to have some comment on this.

We thank the reviewer for raising this important point. We agree that antibody-based labeling introduces a finite linkage error that exceeds the intrinsic localization precision of MINFLUX measurements.

In our experiments, labeling was performed using a primary antibody followed by a secondary nanobody conjugated to a DNA-PAINT docking strand, enabling imaging via transient binding of fluorescent imager strands. This configuration introduces an estimated linkage offset on the order of several nanometers. Importantly, all experimental conditions were labeled using the identical labeling configuration. Therefore, the linkage error is expected to be consistent across conditions and does not affect the relative comparison of pore diameter. While the absolute diameter values may include this systematic offset, the condition-dependent differences remain valid.

We have now explicitly acknowledged this in the revised manuscript (line 130-131 and line 675-677) and clarified that this represents a systematic positional offset relative to the true epitope location.

- Why is a 100x oil immersion used for imaging into water? Z-sclaing was used of 0.67 and the recent Sau Minflux paper is referenced. But this scaling depends on the objective refractive mismatch and the depth of the imaging. Sau et al. argued that this was advice by the manufacturer but is a bit unsatisfactory. S.V. Loginov, D.B. Boltje, M.N.F. Hensgens, J.P. Hoogenboom, and E.B. van der Wee. Depth-dependent scaling of axial distances in light microscopy. Optica, 11(4):553-568, 2024. This could have some impact on lengths estimation along the optical axis - depending how structures are oriented.

We thank the reviewer for raising this important and technically relevant point. We fully agree that refractive index mismatch between oil immersion objectives and aqueous samples leads to depth-dependent axial scaling, as described by Loginov et al., and that this can influence absolute axial distance measurements.

MINFLUX implementations are currently configured with high-NA oil-immersion objectives to achieve the required excitation and detection performance. Water-immersion configurations are not available in the present commercial system. Consequently, axial scaling must be experimentally calibrated for the specific instrument and imaging conditions.

In the work by Sau et al. (DOI: 10.1038/s41586-025-08738-0), the axial scaling factor was not assumed but determined experimentally (see "Measuring the z-scaling factor" and Extended Data Fig. 4) for the specific MINFLUX system used in our study. Our data were acquired on the identical system, with the same objective lens, optical configuration, imaging buffer, and comparable imaging depths. We therefore applied the instrument-specific calibration factor established for this configuration.

We appreciate the reviewer highlighting that this was not explicitly stated in our manuscript; we have now provided a more detailed explanation in the Methods section (line 712-713).

- How was the particle registration done? The given reference to Evangelidis et al. is from 2014. Was their code available and directly applicable to the input data from Minflux as their application was from computer vision and not SMLM? If I remember correctly their code also needed parameters such as the number of Gaussian Mixtures but it is referenced as is?

We thank the reviewer for this important technical question.

The Joint Registration of Multiple Point Clouds (JRMPC) method described by Evangelidis et al. operates on point sets defined by their Cartesian coordinates and can therefore be directly applied to SMLM localization data. While Evangelidis et al. provided a Matlab implementation, at the time we became aware of it, we couldn't make use of it and decided to use an R implementation to better interoperate with our analysis workflows. The R implementation is available in the LOMAR R package (https://cran.r-project.org/package=LOMAR) and is described in Theiss et al. (LOMAR: LOcalization Microscopy Analysis in R; DOI: 10.1101/2022.05.30.493957). Compared to the original method description, this implementation adds a model pruning step based on Figueiredo & Jain (Unsupervised learning of finite mixture models; DOI: 10.1109/34.990138), which makes it less dependent on the initial number of GMM components.

The parameters used in our analysis (tolerance = 1e-5, maximum number of iterations = 100, iterations before starting variance updates = 5, starting number of GMM components = 100, model selection threshold = 0.01) yielded stable and reproducible alignments across independent datasets.

We have clarified the specific implementation in the Methods section ("MINFLUX data analysis", line 737-740) to improve transparency.

Figure 1:

The images 1F show very good labelling density - maybe the best I have seen for Minflux images. How is the data displayed? It would be good to know how many localizations per binding site, NPC are seen on average. This was one of the largest issues with Minflux that the precision per localization is very high, but the images look poor due to missing labels. Here the images look comparable to the best NPC image with STORM/PAINT.

We thank the reviewer for this encouraging assessment of the MINFLUX data quality and for highlighting the importance of labeling efficiency.

The images shown in Fig. 1F display all accumulated single-molecule localizations recorded over the full acquisition period after drift correction and preprocessing. For visualization, 3D localizations were rendered using the MINFLUX data viewer (https://git.embl.de/grp-ic/minflux-data-viewer) in the X-Y or Y-Z view, with axial (Z) positions color-coded and each localization

displayed as an individual scatter point. No additional smoothing was applied; thus, the apparent structural completeness directly reflects the underlying sampling density.

High labeling efficiency was achieved through systematic screening of antibodies and nanobodies against different nucleoporins in combination with a DNA-PAINT labeling strategy. Among the tested reagents, the ELYS antibody provided the most reproducible and structurally complete labeling pattern. On average, we detect a labeling efficiency of 64% ± 3% (mean ± SD; n = 113 NPCs), comparable to previously reported values for Nup96-GFP labeled via GFP nanobody with high labeling efficiency (DOI: 10.1038/s41592-019-0574-9)**.**

Importantly, the combination of MINFLUX with DNA-PAINT provides both high localization precision and repeated transient sampling, allowing each labeled site to be detected multiple times and thereby increasing effective labeling efficiency. This combination mitigates under-sampling and enables reconstruction of structurally more complete NPC rings.

We have clarified the rendering procedure and added quantitative information on the labeling efficiency in the revised Methods section (line 703-707) to ensure transparency.

The shown diameter in the histograms is measured each time by fitting a radius to the correct orientation

We thank the reviewer for this clarification. Yes, each diameter value shown in the histograms was obtained individually for each NPC after 3D alignment.

Point-by-point response (referee comments in blue italics, our response in black)

Reviewer #1 (Remarks to the Author):

The authors have very well addressed all the points I raised, and as far as I can judge - I am not an expert in MD simulations and MINFLUX super-resolution microscopy - the points of the other reviewers.

We thank the reviewer for the highly positive assessment of our revised manuscript and greatly appreciate the thoughtful feedback provided throughout the review process, which has significantly strengthened our study.

Reviewer #2 (Remarks to the Author):

The authors have performed substantial work to revise and improve the manuscript and have adequately addressed the points raised by this reviewer. In my opinion, the revised manuscript fulfils the high-quality standards of NSMB.

We thank the reviewer for this encouraging assessment of our revised manuscript. We greatly appreciate the reviewer's careful evaluation and recognition of the substantial efforts undertaken during the revision process, and are pleased that the revised manuscript now meets the high standards of NSMB.

Reviewer #3 (Remarks to the Author):

The authors carefully and rigorously addressed my main comments on surface tension calculation within solvent free models. I have no further comments.

We thank the reviewer for the positive assessment of our revision and are pleased that the revisions have satisfactorily addressed the concerns raised.

Reviewer #4 (Remarks to the Author):

I co-reviewed this manuscript with one of the reviewers who provided the listed reports. This is part of the Nature Structural & Molecular Biology initiative to facilitate training in peer review and to provide appropriate recognition for Early Career Researchers who co-review manuscripts.

We thank the reviewer and co-reviewer for their careful evaluation of our manuscript.

Reviewer #4 (Remarks on code availability):

n.a.

Reviewer #5 (Remarks to the Author):

Regarding my previously raised concerns the authors have wholeheartedly addressed my concerns and have made substantial improvements to the manuscript. The revisions have significantly strengthened the quality and clarity of the work. From my point of view, the paper is suitable for publication in NSMB now.

We sincerely thank the reviewer for this highly encouraging assessment of our revised manuscript and greatly appreciate the constructive feedback that helped strengthen the quality and clarity of the work.

Reviewer #6 (Remarks to the Author):

The authors have fully addressed my questions for clarifications and also amended them in the manuscript.

NB: Figure 1C, the yz view misses a scale bar.

We thank the reviewer for the positive assessment of our revision and are pleased that the clarifications and corresponding manuscript revisions have satisfactorily addressed the concerns raised.

We thank the reviewer for this observation. The xy and yz views in Fig. 1f share a common scale bar, as is also the case for Fig. 4c. We have clarified this in the corresponding figure legends: "Scale bars: upper panel, 200 nm; lower panel, 500 nm (applies to both x-y and y-z views)."

Reviewer #6 (Remarks on code availability):

Partial. I had a look at the JRMPC R implementation for the fusion.

We thank the reviewer for evaluating the JRMPC R implementation. The code is fully accessible through the repository referenced in the Code Availability statement. We have further verified that the repository contains the complete implementation and documentation required to reproduce the analysis.
